# Sox9 Accelerates Vascular Aging by Regulating Extracellular Matrix Composition and Stiffness

**DOI:** 10.1161/CIRCRESAHA.123.323365

**Published:** 2024-01-05

**Authors:** Maria Faleeva, Sadia Ahmad, Konstantinos Theofilatos, Steven Lynham, Gabriel Watson, Meredith Whitehead, Emilie Marhuenda, Thomas Iskratsch, Susan Cox, Catherine M. Shanahan

**Affiliations:** British Heart Foundation (BHF) Centre of Research Excellence, School of Cardiovascular and Metabolic Medicine & Sciences (M.F., S.A., K.T., G.W., M.W., C.M.S.) King’s College London, United Kingdom.; Proteomics Facility, Centre of Excellence for Mass Spectrometry (S.L.) King’s College London, United Kingdom.; Randall Centre for Cell & Molecular Biophysics, Faculty of Life Sciences & Medicine (S.C.) King’s College London, United Kingdom.; School of Engineering and Material Science, Queen Mary University of London, United Kingdom (E.M., T.I.).

**Keywords:** atherosclerosis, calcium, extracellular matrix, extracellular vesicles, transcription factors

## Abstract

**BACKGROUND:**

Vascular calcification and increased extracellular matrix (ECM) stiffness are hallmarks of vascular aging. Sox9 (SRY-box transcription factor 9) has been implicated in vascular smooth muscle cell (VSMC) osteo/chondrogenic conversion; however, its relationship with aging and calcification has not been studied.

**METHODS:**

Immunohistochemistry was performed on human aortic samples from young and aged patients. Young and senescent primary human VSMCs were induced to produce ECM, and Sox9 expression was manipulated using adenoviral overexpression and depletion. ECM properties were characterized using atomic force microscopy and proteomics, and VSMC phenotype on hydrogels and the ECM were examined using confocal microscopy.

**RESULTS:**

In vivo, Sox9 was not spatially associated with vascular calcification but correlated with the senescence marker p16 (cyclin-dependent kinase inhibitor 2A). In vitro Sox9 showed mechanosensitive responses with increased expression and nuclear translocation in senescent cells and on stiff matrices. Sox9 was found to regulate ECM stiffness and organization by orchestrating changes in collagen (Col) expression and reducing VSMC contractility, leading to the formation of an ECM that mirrored that of senescent cells. These ECM changes promoted phenotypic modulation of VSMCs, whereby senescent cells plated on ECM synthesized from cells depleted of Sox9 returned to a proliferative state, while proliferating cells on a matrix produced by Sox9 expressing cells showed reduced proliferation and increased DNA damage, reiterating features of senescent cells. LH3 (procollagen-lysine, 2-oxoglutarate 5-dioxygenase 3) was identified as an Sox9 target and key regulator of ECM stiffness. LH3 is packaged into extracellular vesicles and Sox9 promotes extracellular vesicle secretion, leading to increased LH3 deposition within the ECM.

**CONCLUSIONS:**

These findings highlight the crucial role of ECM structure and composition in regulating VSMC phenotype. We identify a positive feedback cycle, whereby cellular senescence and increased ECM stiffening promote Sox9 expression, which, in turn, drives further ECM modifications to further accelerate stiffening and senescence.

**Novelty and Significance**

**What Is Known?**

- Vascular smooth muscle cells (VSMCs) differentiate into an osteo/chondrogenic phenotype during vascular calcification and aging.
- VSMCs alter the composition of the extracellular matrix (ECM) during vascular aging.
- Sox9 (SRY-box transcription factor 9) is a strong regulator of chondrocytic differentiation and ECM-related gene expression.

**What New Information Does This Article Contribute?**

- Sox9 drives the senescent ECM phenotype, impacting stiffness, organization, and protein composition.
- Senescent ECM promotes VSMC DNA damage and exit from the cell cycle, while young ECM promotes senescent VSMCs to reenter the cell cycle.
- Sox9 and cellular senescence upregulate LH3 (procollagen-lysine, 2-oxoglutarate 5-dioxygenase 3) deposition in the ECM through extracellular vesicles increasing ECM stiffness.

Vascular calcification is a prevalent, detrimental aging pathology. Sox9 has previously been identified as a key regulator of VSMC osteo/chondrogenic differentiation. This study shows that Sox9 is increased in vascular aging where it promotes compositional and structural ECM changes that mimic features of vascular aging. Healthy VSMCs exposed to Sox9-modified ECM show accelerated senescence, while senescent cells are partially rejuvenated when exposed to Sox9-depleted ECM. These novel findings highlight an underappreciated role of the ECM in regulating VSMC aging and identify key factors including LH3 as drivers of VSMC stiffening with age.

**In This Issue, see p 245**

**Meet the First Author, see p 246**

**Editorial, see p 325**

Vascular calcification is a life-threatening pathology that is strongly associated with vascular aging. It is caused by the abnormal deposition of calcium salts in the vessel intima and media and contributes to the development of atherosclerosis, as well as arterial stiffening.^1^ Vascular calcification is a cell-mediated process associated with the modulation of vascular smooth muscle cells (VSMCs) to an osteo/chondrogenic phenotype characterized by loss of contractile markers,^2,3^ increased expression of key TFs (transcription factors) associated with developmental osteo/chondrogenesis,^4,5^ and increased extracellular vesicle (EV) release.^6,7^

The transition of VSMCs to an osteo/chondrogenic phenotype has been tightly linked with cellular aging. Senescent VSMCs are increased in the calcified vessel wall and calcify more readily in vitro.^8–10^ Senescent cells are also proinflammatory, expressing a senescence-associated secretory phenotype, releasing cytokines, chemokines, and interleukins, as well as EVs.^11^ Secreted EVs are involved in both physiological and pathological processes, mediating intracellular communication but also depositing in the extracellular matrix (ECM) to form a nidus for mineralization.^12^ During senescence, there are changes in cargo loading of EVs, resulting in an increased abundance of calcification-promoting factors. Furthermore, these EVs have the potential to modulate the phenotype of neighboring cells.^13^

In addition to mineralization, the aging vasculature also undergoes dramatic changes in ECM composition and structure that contribute to vascular stiffening. In the aorta, there is a decrease in elastin deposition and an increase in its fragmentation, increased collagen (Col) deposition, and an increase in the crosslinking of Col fibers, with some of these changes also implicated in promoting calcification.^14,15^ Many of these ECM changes have been attributed to wear and tear or oxidation processes or have been studied in the context of disease. Few studies have considered how VSMC aging and senescence may act to directly modulate ECM aging or to establish the factors that might regulate this interplay. Indeed, ECM proteins play a dual role in regulating both the integrity of the vasculature and extracellular cell signaling, yet how ECM aging impacts on VSMC phenotype remains poorly understood.

Runx2 (RUNX family transcription factor 2) and Sox9 (SRY-box transcription factor 9) are master regulators of bone and cartilage differentiation. These TFs cooperate to orchestrate the expression of numerous matrisomal proteins required to form and promote, or not, the mineralization of these specialist tissues.^16^ Both are also expressed in the calcified vasculature.^5^ However, while the role of Runx2 in activating the expression of osteogenic genes during vascular calcification and aging has been well characterized,^17–20^ less is known about the expression and role of Sox9. Interestingly, Sox9 plays a key role during vascular development. In sclerotomal progenitor cells, Sox9 is vital for cell fate specification between VSMCs and chondrocytes and must be silenced via notch signaling^21^ to enable VSMC differentiation. This silencing allows derepression of myocardin, a key TF regulating expression of smooth muscle marker genes, such as *TAGLN* and *ACTA* encoding α-smooth muscle actin (α-SMA). Sox9 also induces VSMC dedifferentiation in vitro by decreasing the expression of contractile genes.^22–24^ On the other hand, maintenance of Sox9 expression during development facilitates the expression of numerous ECM genes, such as *Collagen2*, *Collagen9*, and *Collagen11*, essential for chondrocyte differentiation.^25,26^ Vascular injury and aging can prompt a decrease in notch signaling^27^ and potentially induce reexpression of Sox9.^21,23^ Expression of Sox9 has been demonstrated in mouse models of arteriosclerosis where it has been shown to play roles in ECM reorganization through its upregulation of *Collagen 2*,^23^ as well as in calcification,^23^ via the regulation of *PRG4* (proteoglycan 4).^28^ In the human vasculature, Sox9 expression has been shown to occur in aged and calcified aortic tissue^5^; however, its role in ECM remodeling and calcification has been largely overlooked.

In this study, we describe a novel role for Sox9 in human vascular aging. We show that Sox9 expression correlates with VSMC senescence and demonstrate that Sox9 shows mechanosensitive responses in aged VSMCs. We also show that it is a critical mediator of ECM stiffness through activation of the Col modifier LH3 (procollagen-lysine, 2-oxoglutarate 5-dioxygenase 3), and its role in driving increased secretion of LH3 in EVs. Importantly, the ECM produced in response to Sox9 mimics key features of the senescent ECM and acts as a potent inhibitor of VSMC proliferation while concomitantly driving DNA damage and inflammation to accelerate VSMC aging.

## METHODS

### Data Availability

The mass spectrometry proteomics data have been deposited to the ProteomeXchange Consortium via the Proteomics Identification Database (PRIDE)^29^ partner repository with the dataset identifiers PXD046001 and 10.6019/PXD046001. All supporting data are available within the article and its Supplemental Material. The Expanded Materials and Methods section is given in Supplemental Material. Research materials listed in the Methods section are included in Table S1.

## RESULTS

### Sox9 Expression in the Vessel Media Correlates With Loss of Contractile Markers and Aging but Not Calcification

**Figure 1.**
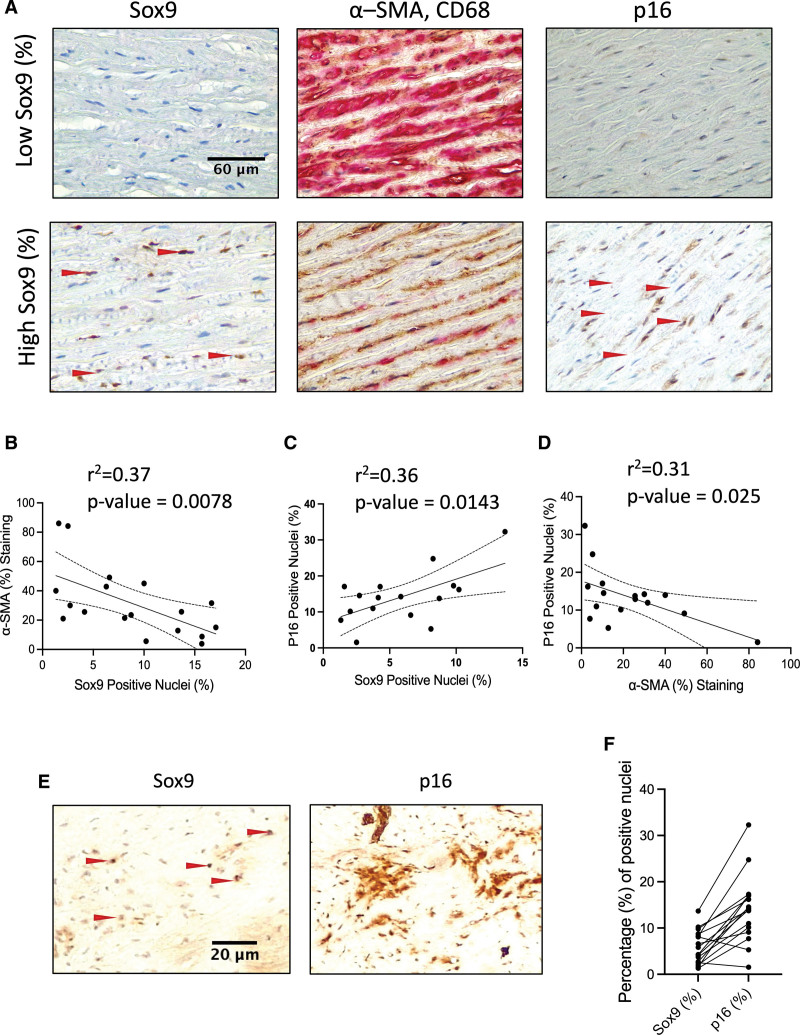
**Sox9 (SRY-box transcription factor 9) expression in vascular smooth muscle cells (VSMCs) correlates with increased cellular senescence and decreased expression of α-smooth muscle actin (α-SMA). A**, Sox9, α-SMA, Cluster of Differentiation 68 (CD68), and p16 (cyclin-dependent kinase inhibitor 2A) staining in the medial layer of human aortic samples. **B**, Correlation of Sox9 positive nuclei with α-SMA (n=18), (**C**) p16 positive nuclei (n=16), and (**D**) α-SMA with p16 positive nuclei (n=16). Normality was validated via the Shapiro-Wilk test. Correlation was performed using the Pearson test. **E**, Immunohistochemistry of serial sections stained for Sox9 and p16. Red arrows highlight positively stained nuclei. **F**, Bar graph showing percentages of Sox9 and p16 nuclei in each sample.

We performed immunohistochemistry on human aortic tissue samples from young and old patients with and without vascular calcification. Nuclear Sox9 expression was detectable in the vessel media predominantly in VSMCs (Figure 1A). As expected, there was a strong correlation between patient age and calcification, and age and expression of p16 (cyclin-dependent kinase inhibitor 2A), a marker of cell senescence (Figure S1A through S1C). However, we found no association between Sox9 expression and calcification (Figure S1D and S1E). We observed some noncalcified areas with high levels of Sox9, while areas of high calcification were negative for Sox9 staining (Figure S1A). Using an in vitro calcification assay, we verified that calcification propensity was not increased in Sox9 overexpressing VSMCs, suggesting that there is not a direct relationship between Sox9 and mineralization in human cells (Figure S1F and S1G). However, we did find a correlation between Sox9 and the senescence marker p16. Elevated expression of Sox9 was also associated with decreased α-SMA expression (Figure 1B and 1C) with p16 also showing the same negative correlation (Figure 1D). Immunohistochemistry of Sox9 and p16 in serial sections revealed more p16 positive cells than Sox9 positive cells in any given section, with the majority of Sox9 positive nuclei also staining positive for p16, typically in areas that were cell-poor and matrix-rich (Figure 1E and 1F). This suggested that senescent VSMCs may be providing an environment that promotes elevated Sox9 expression.

### Sox9 Shows Mechanosensitive Responses in Senescent VSMCs

**Figure 2.**
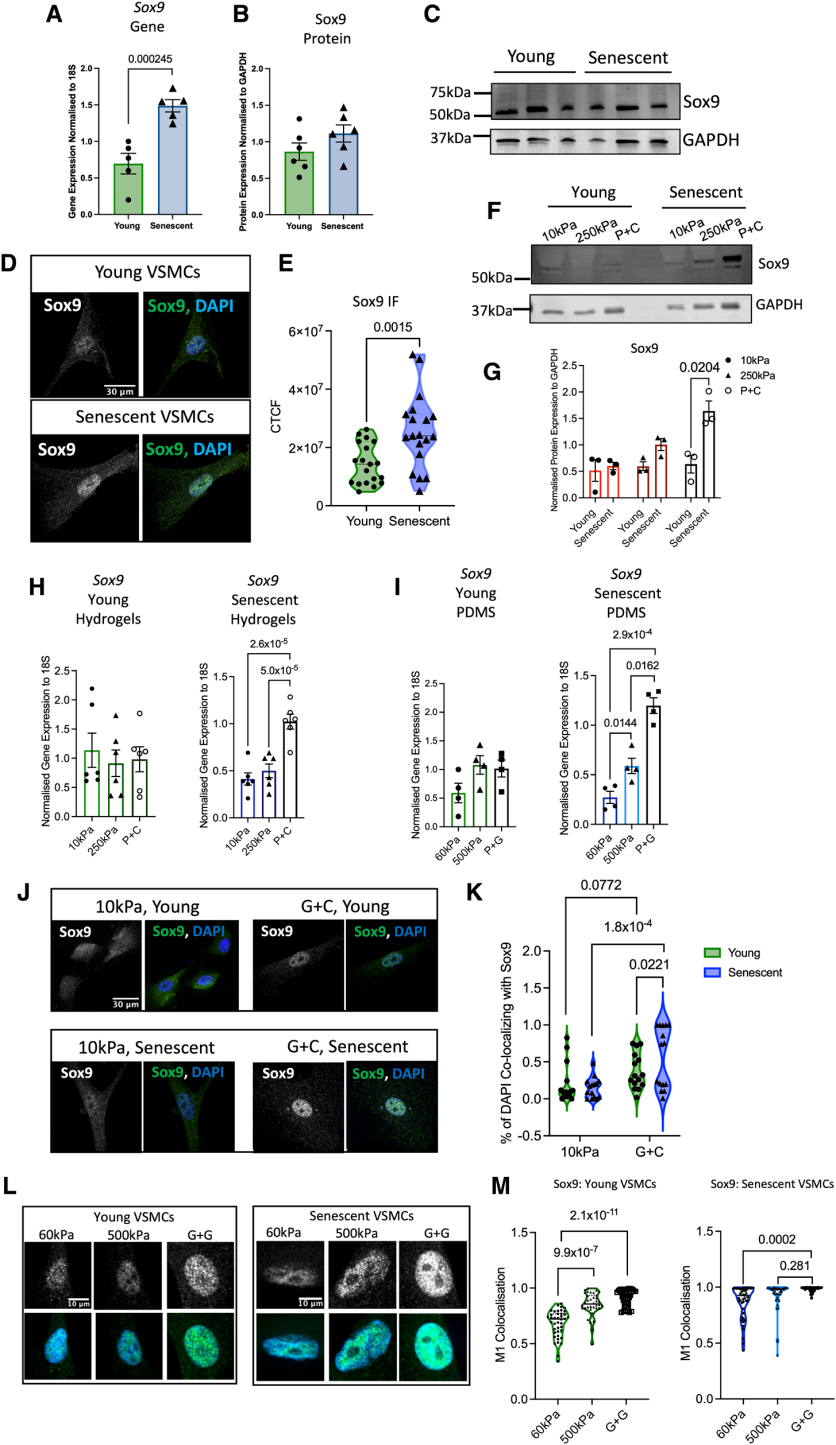
**Sox9 (SRY-box transcription factor 9) is mechanosensitive in senescent vascular smooth muscle cells (VSMCs). A** through **C**, *Sox9* gene and protein expression in young and senescent VSMCs (n=5, 6 from 3 isolates). Normality was confirmed with the Shapiro-Wilk test; 2-way ANOVA, false rate discovery (FDR) correction, and q-values are shown. **D** and **E**, Immunofluorescence (IF) in young and senescent VSMCs and quantification of Sox9 corrected total cell fluorescence (CTCF; n=18 from 3 isolates). Normality was confirmed with the Shapiro-Wilk test; 2-way ANOVA, FDR correction, and q-values are shown. **F** and **G**, Sox9 protein expression in young and senescent VSMCs on hydrogels and collagen-coated plastic (P+C; n=3). Normality was confirmed with the Shapiro-Wilk test; 2-way ANOVA, FDR correction, and q-values are shown. **H**, *Sox9* gene expression in young and senescent VSMCs on different substrates (n=6 technical repeats from 3 isolates) and (**I**) 60- and 500-kPa Poly(dimethylsiloxane) (PDMS) gels and gelatin-coated plastic (P+G; n=4 technical repeats from 3 isolates). Normality was confirmed via the Shapiro-Wilk test; 2-way ANOVA, FDR correction, and q-values are shown. **J** through **M**, Quantification of Sox9 nuclear colocalization and IF in young and senescent VSMCs on different substrates (**J** and **K**) 10- and 250-kPa hydrogels and glass coated with collagen (G+C; n=15 technical repeats from 3 isolates) and (**L** and **M**) 60- and 500-kPa PDMS gels and glass coated with gelatin (G+G; n=45 technical repeats from 3 isolates). Sox9 is shown in gray and green, and nuclear 4’,6-diamidino-2-phenylindole (DAPI) staining is shown in blue. Normality was rejected via the Shapiro-Wilk test; mixed-effects analysis, FDR correction, and q-values are shown. GAPDH indicates glyceraldehyde-3-phosphate dehydrogenase.

The association between p16 and Sox9 in vivo led us to investigate whether Sox9 is increased during cellular senescence. We cultured primary human VSMCs to replicative senescence using serial passaging and found that senescent VSMCs showed increased expression of Sox9 at the RNA level, but not consistently at the protein level (Figure 2A through 2C). Immunofluorescence, however, showed more nuclear localization of Sox9 in senescent VSMCs compared with their early passage counterparts (Figure 2D and 2E). To investigate whether Sox9 can promote senescence, Sox9 was overexpressed in the early passage (hereafter termed young) VSMCs using adenoviral transduction (Sox9 overexpression). This resulted in a decrease in contractile markers such as α-SMA consistent with its role in regulating VSMC differentiation (Figure S2A through S2C). Analysis of *p16* gene expression showed that it was upregulated in response to Sox9 overexpression and in senescent VSMCs compared with young controls (Figure S2D and S2F). However, Sox9 depletion (Sox9 knockout) in senescent cells had no effect on *p16* (Figure S2D through S2F) suggesting additional factors are involved in Sox9 regulation during VSMC aging.

In chondrocytes, Sox9 is mechanosensitive and shows increased expression in stiff environments^30,31^; therefore, we tested whether Sox9 might also be mechanosensitive in VSMCs. Young and senescent VSMCs were plated onto Col-coated plastic and hydrogels of 10- or 250-kPa stiffness. While young VSMCs showed no changes in *Sox9* gene expression, senescent VSMCs markedly increased expression on stiff plastic at both the gene and protein levels (Figure 2F through 2H). To explore further the mechanosensitive responses of Sox9, we next used Poly(dimethylsiloxane) (PDMS) gels to enable a greater range of stiffnesses. We found that stiffnesses ranging from 1 to 60 kPa did not result in modulation of *Sox9* at the gene level (Figure S2G and S2H). However, senescent VSMCs again displayed an upregulation of *Sox9* from soft 60 kPa to stiff plastic. This prompted us to explore whether the mechanosensitive responses of Sox9 were only prevalent on stiffer surfaces. To this end, 500-kPa PDMS gels were synthesized, and the expression of Sox9 was evaluated via qPCR and immunofluorescence. At this stiffness, *ACTA2* showed mechanosensitivity as expected (Figure S2I); however, young VSMCs again did not exhibit any change in *Sox9* expression. In contrast, Sox9 increasingly scaled in expression when senescent VSMCs were plated on 60 kPa, 500 kPa, and plastic (Figure 2I).

Immunofluorescence revealed that these changes in gene expression in response to stiffness were accompanied by changes in the cellular localization of Sox9. On soft matrices of 10 or 60 kPa, Sox9 was diffusely localized in both the cytoplasm and nucleus (Figure 2J and 2K). This was most pronounced in young cells with senescent cells already showing nuclear localization. When plated on stiff matrices, such as Col- or gelatin-coated plastic or 500-kPa gels, Sox9 translocated into the nucleus in both young and senescent cells (Figure 2L and 2M). Quantification revealed augmentation of colocalization of Sox9 with 4’,6-diamidino-2-phenylindole (DAPI) in the nucleus in senescent VSMCs compared with their young counterparts and on stiff matrix compared with soft. Nuclear translocation indicates a potential increase in Sox9 transcriptional activity. This was confirmed using reverse transcription-quantitative PCR (RT-qPCR) for *Sox5* and *Sox6*, 2 transcriptional targets of Sox9, which also showed increased expression on stiff surfaces (Figure S2J through S2M).

### Sox9 Regulates VSMC ECM Organization and Stiffness

**Figure 3.**
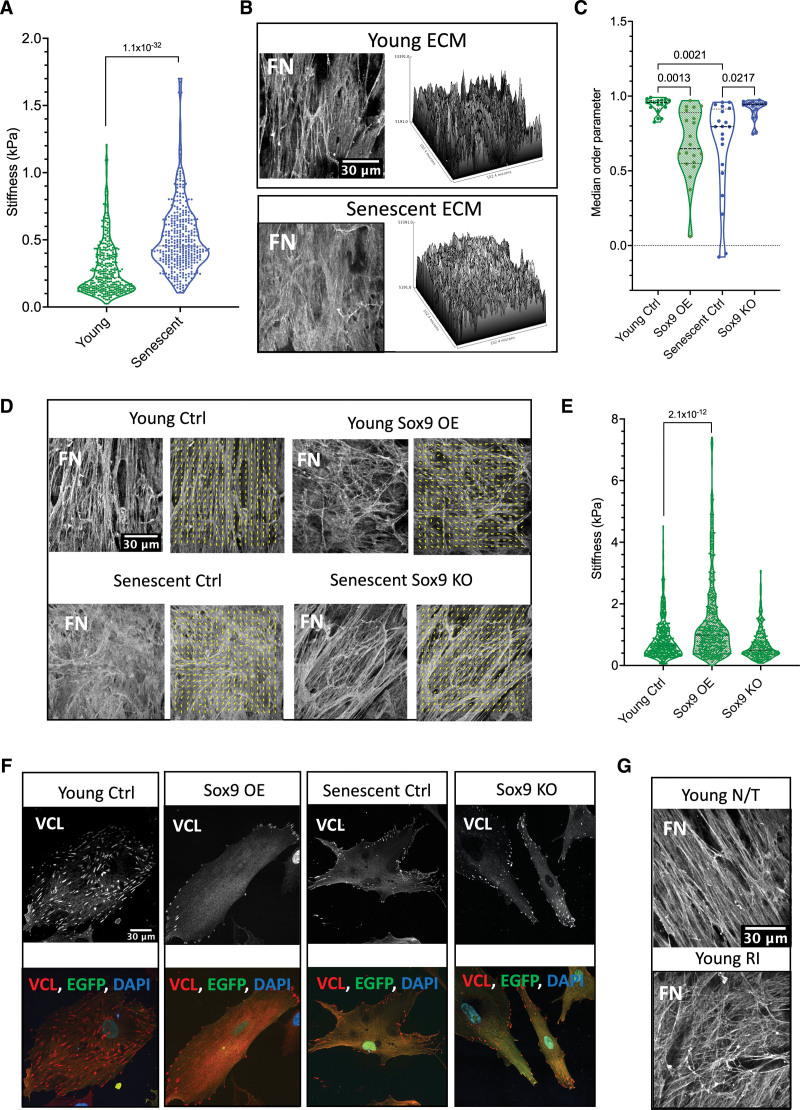
**Vascular smooth muscle cell (VSMC) senescence and Sox9 (SRY-box transcription factor 9) expression regulate the extracellular matrix (ECM). A**, Atomic force microscopy (AFM) stiffness measurements of ECM synthesized from young (n=352 from 3 isolates) and senescent (n=354 from 3 isolates) VSMCs. Normality was rejected via the Shapiro-Wilk test; linear mixed-effect analysis and *P* value are shown. **B**, Representative fibronectin immunofluorescence (IF) staining and topology of young and senescent ECM. **C**, Median order parameter of analyzed fibronectin fiber alignment and (**D**) representative IF staining of ECM (n=20) control (Ctrl), overexpression (OE), and knockout (KO). Normality was rejected via the Shapiro-Wilk test; mixed-effect analysis, multiple testing correction via the false rate discovery (FDR) method of Benjamini and Hochberg, and q-values are presented. Fiber alignment is indicated by the yellow lines. **E**, AFM measurements of ECM synthesized from young VSMCs transfected with EGFP (enhanced green fluorescent protein; young Ctrl; n=532 from 3 isolates), Sox9 overexpression (Sox9 OE; n=448 from 3 repeats), and knockout (Sox9 KO; n=228 from 3 isolates) adenovirus. Normality was rejected via the Shapiro-Wilk test; mixed-effect analysis, multiple testing correction via the FDR method of Benjamini and Hochberg, and q-values are presented. **F**, Representative IF of VCL (vinculin) in young Ctrl, Sox9 OE, senescent Ctrl, and Sox9 KO. VCL is shown in grey/red, EGFP in green, and nuclear staining (4’,6-diamidino-2-phenylindole [DAPI]) in blue. **G**, Representative IF of fibronectin in ECM synthesized from young VSMCs with no treatment (young non-treated [N/T]) and young VSMCs treated with rock inhibitor (young Rho-associated protein kinase [ROCK] Inhibitor [RI]). FN indicates fibronectin.

Sox9 can regulate an array of ECM-related genes in chondrocytes^26^ and mouse VSMCs.^23^ Thus, we wondered whether Sox9 may regulate ECM changes during vascular aging. To examine the effects of aging on matrix stiffness, ECM was synthesized from young and senescent VSMCs, the cells were removed, and the stiffness of the native ECM was measured by atomic force microscopy. This revealed an increase in the stiffness of ECM produced by senescent VSMCs compared with that of young cells (Figure 3A). Analysis of the ECM by immunofluorescence with fibronectin staining revealed that ECM synthesized by young cells showed an organized fibrillar pattern. In contrast, the senescent ECM showed a woven, matted appearance with disorganized fibers. Alignment by the Fourier transform analysis confirmed quantitative differences in the matrix alignment (Figure 3B through 3D). The z-stack analysis showed no significant differences between the thickness of the ECM produced by the VSMCs (young 8.65 mm±0.55 SEM and senescent 8.75 mm±0.325 SEM); however, there were visible differences in the topology of the fibronectin staining. The young ECM showed prominent tall peaks with deep valleys, while the senescent ECM was comparatively flatter with minimal variations in height between the peaks and valleys.

To investigate the potential role of Sox9 in regulating ECM stiffness and fiber organization, Sox9 was overexpressed or depleted in young and senescent VSMCs, respectively, and the resulting ECM was analyzed (Figure 3C through 3E). Sox9 overexpression led to an increase in ECM stiffness in young cells and a decrease in parallel fiber alignment, creating an ECM resembling that produced by senescent VSMCs. Conversely, its depletion in senescent cells resulted in decreased stiffness and an increase in parallel fibers quantitatively no different from the alignment observed in young ECM (Figure 3C and 3D). To understand the mechanism behind this, we tested whether Sox9 regulates fiber alignment through focal adhesions (FAs), which anchor cells to the ECM. Overexpression of Sox9 resulted in a reduction and redistribution of vinculin, a marker for FAs.^32^ FAs were present over the entire cell area in young control cells but only present around the perimeter of the cell membrane in response to Sox9 reminiscent of the FA localization observed and previously described in senescent VSMCs.^32^ Depletion of Sox9 in senescent cells led to a visible increase and a redistribution away from the cellular periphery of vinculin FAs but was insufficient to fully return the FAs back to their original density as seen in young VSMCs (Figure 3F). Importantly, we could replicate the disorganized fiber alignment found in senescent or Sox9-expressing VSMCs by treating young VSMCs with an ROCK (Rho-associated protein kinase) inhibitor to reduce cellular contractility to mimic the effect of Sox9 on reducing VSMC contraction (Figure 3G). These findings suggest that Sox9 plays a crucial role in regulating ECM stiffness and fiber organization in part via regulating cellular contractility and FA localization.

### Sox9-Regulated ECM Regulates VSMC Phenotype

**Figure 4.**
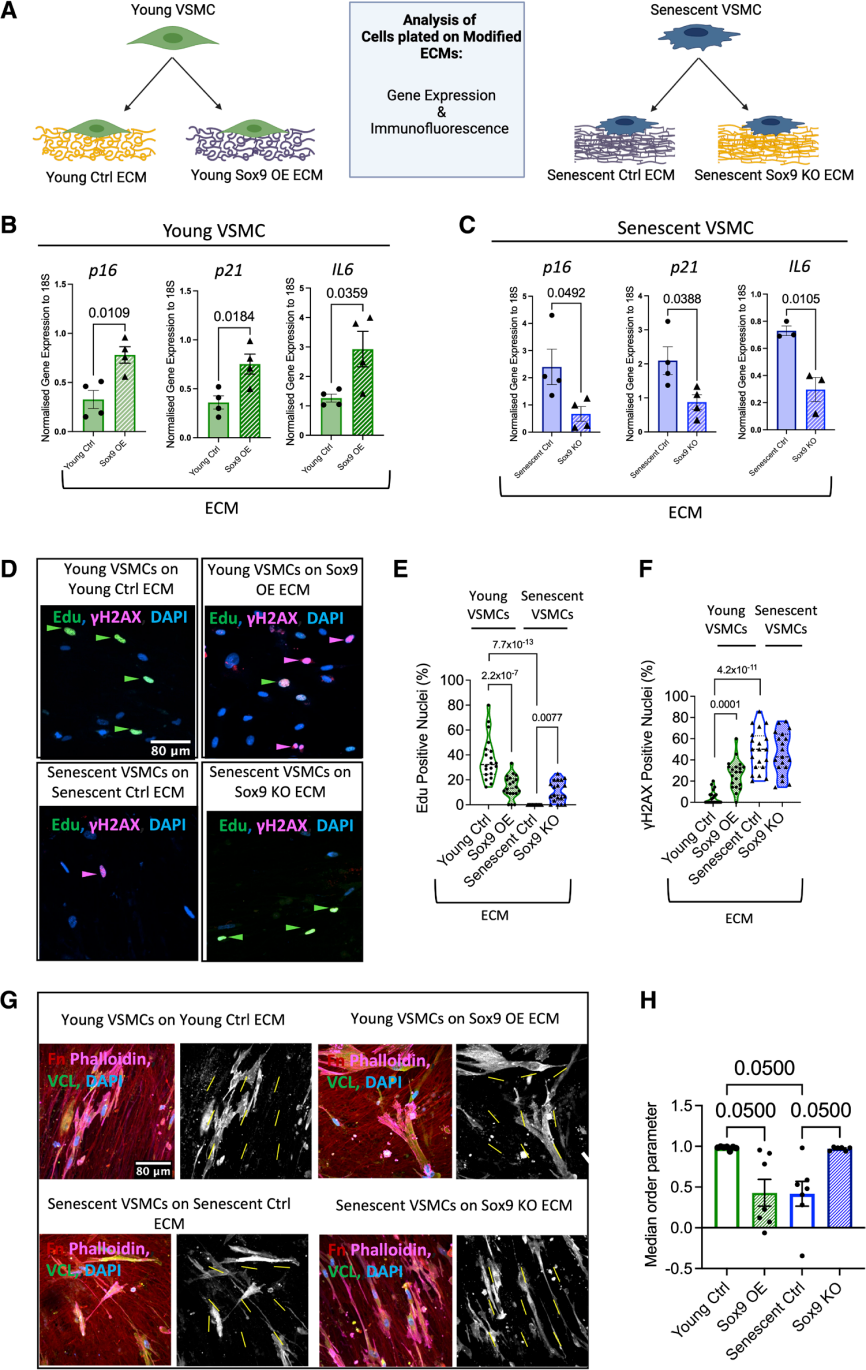
**Sox9 (SRY-box transcription factor 9)-modified extracellular matrix (ECM) regulates vascular smooth muscle cell (VSMC) phenotype. A**, Schematic of experimental protocol. Reverse transcription-quantitative PCR (RT-qPCR) of p16 (cyclin-dependent kinase inhibitor 2A), p21 (cyclin-dependent kinase inhibitor 1), and IL6 (interleukin 6) from (**B**) young (n=4 from 3 isolates) and (**C**) senescent VSMCs (n=4/3 from 3 isolates) plated on transduced ECMs. Normality was validated via the Shapiro-Wilk test and the unpaired Student *t* test. **D**, Representative immunofluorescence (IF) and quantification (%) of (**E**) 5-ethynyl-2′-deoxyuridine (EdU) and (**F**) γH2AX (phosphorylation of the Ser-139 residue of the histone variant H2AX) from VSMCs plated on transduced ECMs. EdU, γH2AX, and nuclear 4’,6-diamidino-2-phenylindole (DAPI) staining are shown in green, magenta, and blue, respectively (n=20). Normality was validated via the Shapiro-Wilk test; 2-way ANOVA and q-values adjusted for multiple testing with Benjamini-Hochberg FDR correction are shown. **G**, Representative IF staining of young and senescent VSMCs plated on transduced ECMs with Fn (fibronectin) in red, phalloidin in magenta, VCL (vinculin) in green, and nuclear staining (DAPI) in blue. Adjacent black and white images of vinculin staining with yellow lines indicating cellular alignment within the frame. **H**, Quantified median order parameter of cellular alignment of young and senescent VSMCs plated on ECM synthesized from young Ctrl (n=7), Sox9 overexpression (OE; n=7), senescent Ctrl (n=7), and Sox9 knockout (KO; n=6) VSMCs (taken from 3 isolates). Normality was rejected via the Shapiro-Wilk test; mixed model analysis and q-values adjusted for multiple testing with Benjamini-Hochberg false rate discovery (FDR) correction are shown.

We next investigated whether the Sox9-modulated ECM could influence VSMC phenotype and senescence. We again synthesized ECM from young and senescent VSMCs with Sox9 overexpression or depletion, respectively, and replated fresh young or senescent VSMCs onto their corresponding decellularized ECM (Figure 4A). Young VSMCs plated on ECM synthesized from young VSMCs overexpressing Sox9 had increased expression of *p16* and *p21*, as well as elevated *IL6*, an inflammatory senescence-associated secretory phenotype marker,^33^ compared with cells plated on EGFP (enhanced green fluorescent protein) control young ECM. In contrast, senescent cells plated on matrices synthesized from senescent VSMCs depleted of Sox9 exhibited downregulation of both *p16* and *p21*, as well as *IL6* (Figure 4B and 4C). Importantly, cell proliferation, measured via 5-ethynyl-2′-deoxyuridine (EdU) incorporation, and DNA damage analysis were consistent with the changes in expression of these senescent cell cycle and inflammatory markers observed in cells plated on Sox9-modified matrices. Young VSMCs plated on young Sox9 overexpressing ECM had reduced cell proliferation and an increased number of DNA double-strand breaks shown by γH2Ax (phosphorylation of the Ser-139 residue of the histone variant H2AX) foci consistent with DNA damage driving cell cycle arrest and senescence. Conversely, senescent cells plated on senescent Sox9 knockout ECM were able to reenter the cell cycle as shown by increased EdU incorporation without any change in DNA damage (Figure 4D through 4F). Furthermore, we observed that the orientation of the VSMCs was highly dependent on the ECM. Both young and senescent VSMCs when plated on young control and senescent Sox9 knockout matrices exhibited parallel orientation to one another (Figure 4G and 4H). VSMCs plated on young Sox9 overexpression and senescent control ECM exhibited random orientation, frequently aligning at perpendicular angles with each other (Figure 4G and 4H). This change in cell orientation is likely due to a lack of fibronectin fiber definition and alignment. To rule out that the changes in gene expression observed were due to changes in matrix stiffness, we performed RT-qPCR for *p16*, *p21*, and *IL6* on hydrogels of different stiffness. None were mechanosensitive (Figure S3A through S3F), while expression of the mechanosensitive genes, *ACTA* and *TAGLN*, which are reduced on soft matrices, did not show any changes in response to Sox9-mediated ECM modulation (Figure S3I through S3L). This suggests that it is ECM composition, not stiffness or alignment, which is driving these changes in gene expression.

### Sox9 Regulates Protein Ratios Deposited in the ECM

**Figure 5.**
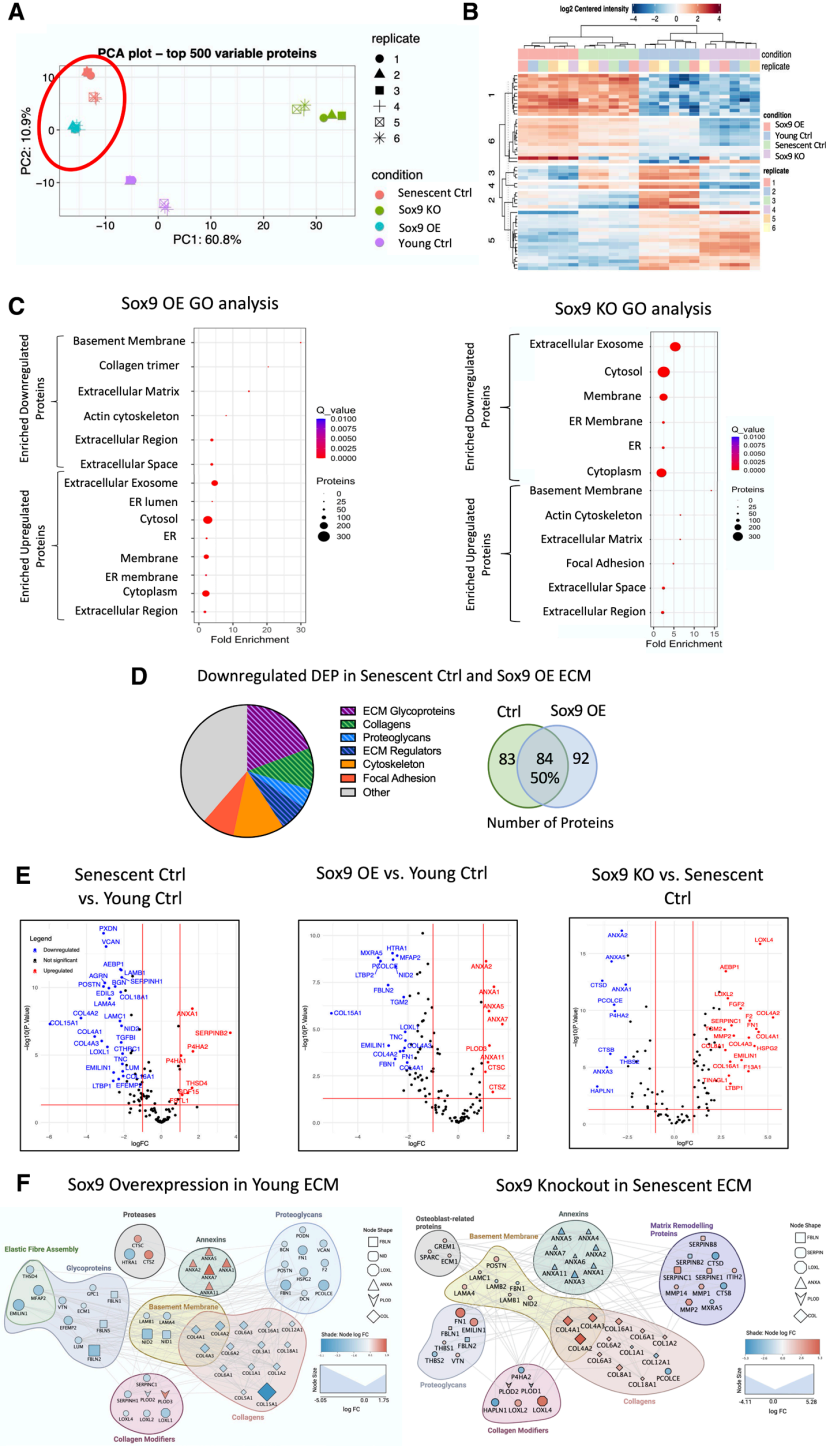
**Sox9 (SRY-box transcription factor 9) drives extracellular matrix (ECM) composition toward a senescent-like phenotype (n=6 injections for each condition from a 35-year-old female and a 38-year-old female). A**, Principle component analysis (PCA) plot of ECM samples synthesized from young vascular smooth muscle cells (VSMCs) treated with either EGFP (enhanced green fluorescent protein) control (young Ctrl) or Sox9 overexpression adenovirus (Sox9 overexpression [OE]) and senescent VSMCs treated with either short hairpin EGFP (shEGFP) control adenovirus (senescent Ctrl) or Sox9 knockout (KO) adenovirus. **B**, Heatmap of clustered differentially expressed proteins (DEPs), showing similarity between ECM synthesized from young Ctrl and Sox9 KO and between Sox9 OE and senescent Ctrl. **C**, Gene ontology (GO) analysis of DEPs in Sox9 OE ECM compared with young Ctrl and Sox9 KO compared with senescent Ctrl. **D**, Pie chart and Venn diagram of downregulated DEP in both Sox9 OE and senescent Ctrl ECM. **E**, Volcano plots of DEP comparing senescent Ctrl against young Ctrl, Sox9 OE against young Ctrl, and Sox9 KO against senescent Ctrl. **F**, Protein-protein interaction network showing interactions between significantly upregulated and downregulated proteins in Sox9 OE ECM compared with the young Ctrl ECM and in Sox9 KO ECM compared with the senescent Ctrl ECM. ER indicates endoplasmic reticulum.

To gain insights into the specific components of the ECM, which are directly regulated by Sox9, we conducted mass spectrometry analysis on young and senescent decellularized matrix with and without Sox9 overexpression and knockout, respectively (Figure 5). Principle component analysis and differentially expressed protein clustering showed young and senescent ECM formed distinct clusters (Figure 5A and 5B). Notably, ECM from young Sox9 overexpression clustered most closely with senescent control ECM, while senescent Sox9 knockout ECM formed a separate cluster more closely related to young control ECM, as illustrated by the heatmap (Figure 5B). Gene ontology analysis first comparing young and senescent control ECM showed the main pathways downregulated were FA, basement membrane, and ECM, while upregulated pathways included extracellular exosome (Figure S4A). Remarkably, we found similar pathway changes when Sox9 overexpression ECM was compared with young control ECM with a decrease in ECM-related proteins and an increase in proteins associated with extracellular exosomes and cytoplasmic proteins. The opposite was observed when senescent Sox9 knockout ECM was compared with senescent ECM, which showed an increase in ECM-related proteins and a decrease in exosome and cytoplasmic components (Figure 5C). Further comparative analysis to determine whether the proteins changing within the ECM were similar showed there was a 50% overlap in the differentially expressed proteins between senescent control and young Sox9 overexpression ECM when both were compared independently with the young control ECM. The majority of these proteins were glycoproteins and Cols in the matrisomal protein group (Figure 5D). Volcano plots highlighted Col15a1 as having the highest negative log fold change in both the senescent control and the young Sox9 overexpression ECM compared with the young control ECM (Figure 5E). Other matrisomal proteins that were reciprocally changed in each group and also differentially expressed between young and senescent control ECM included *Collagen 4* and *EMILIN1* (elastin microfibril interfacer 1). The senescent Sox9 knockout ECM also exhibited a strong upregulation of *LOXL4* (lysyl oxidase-like 4), a key modulator of Col and elastin cross-links (Figure 5E). There were also reciprocal changes in several Anx (annexin) proteins that are abundant in EV cargoes.^34^

To further understand the functional relationships between the differentially expressed matrisomal proteins regulated by Sox9, we constructed protein-protein interaction networks. This revealed that the most downregulated proteins in the young Sox9 expression ECM were glycoproteins and Cols. Among these were a group of proteins associated with elastic fiber assembly and deposition including EMILIN1 and FBLN 1/2 (fibulins 1/2). There was also a clear subset of basement membrane components, such as collagens 4a1-2 (*Col4a1-2*) and 6 (*Col6a1-2*) and Nidogens 1/2 (NID1/2). Col modifier proteins including LOXL2/4 were mostly downregulated; however, 2 modifiers, CTSC (cathepsin C) and LH3/*Plod3*, were upregulated. A large subset of Anx proteins including those enriched in VSMC EVs were also upregulated.^34^ Conversely, ECM synthesized from senescent VSMCs depleted of Sox9 showed the opposite changes. There was clear upregulation of the same elastin modifiers, as well as Cols and basement membrane proteins. ECM-remodeling proteins were also upregulated including LOXL4 (Figure 5F). Interestingly, both PLOD1/2 were downregulated as were Anxs. We did not see any changes in key chondrocyte Sox9 targets, such as *Collagen 2* and *Collagen 11*. We validated the changes in basement membrane components using RT-qPCR and immunofluorescence that revealed clear defects in basement membrane assembly of Col 4 in both senescent and young Sox9 expressing VSMCs, further highlighting the key role Sox9 plays in regulating these protein networks (Figure S4B through S4E).

### Sox9 Regulates the Posttranslational Modification of Col Fibrils via LH3

**Figure 6.**
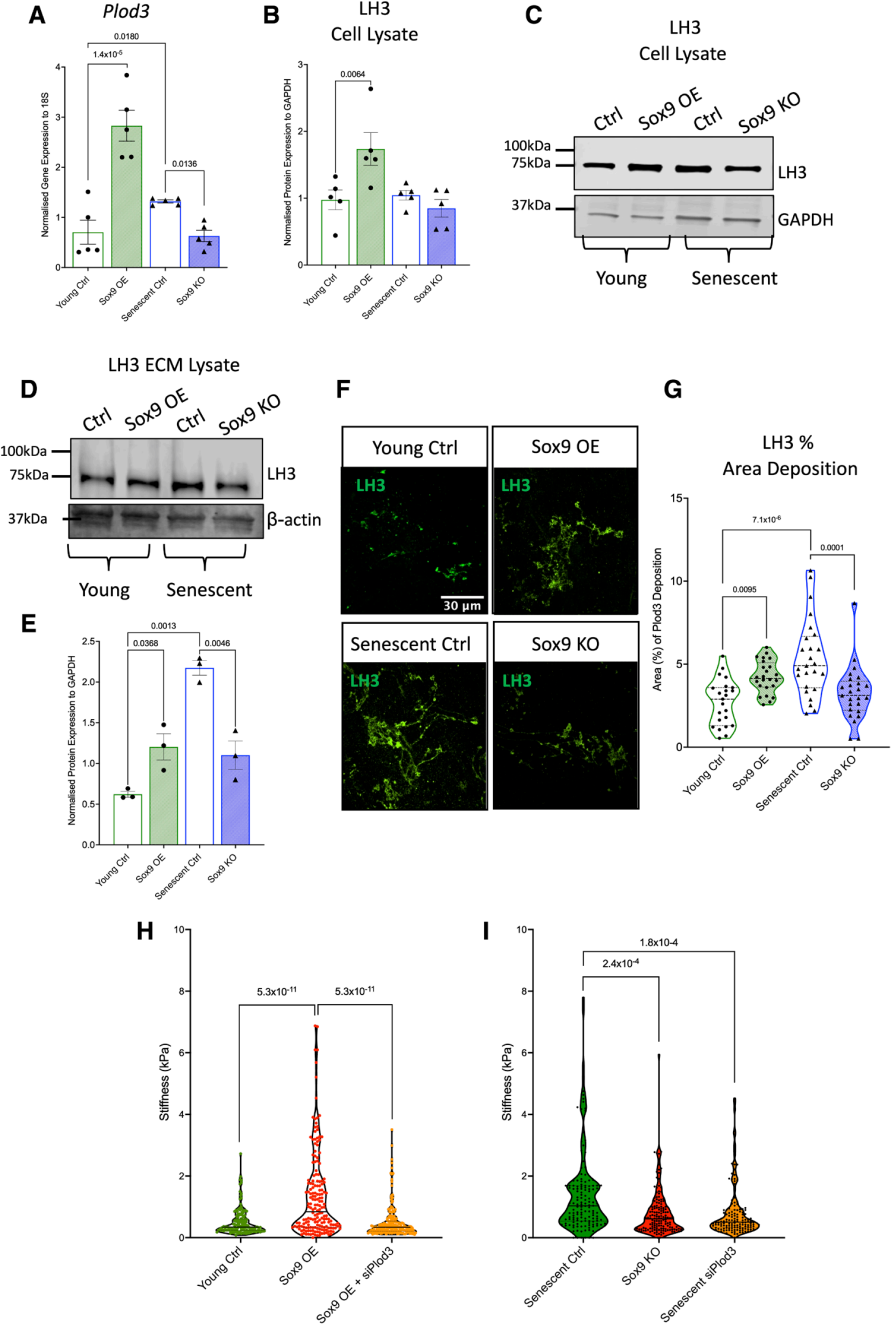
***Plod3*/LH3 (procollagen-lysine, 2-oxoglutarate 5-dioxygenase 3) expression and deposition into the extracellular matrix (ECM) is regulated by Sox9 (SRY-box transcription factor 9). A**, Reverse transcription-quantitative PCR (RT-qPCR) quantification of *Plod3* expression in young vascular smooth muscle cells (VSMCs) transduced with EGFP (enhanced green fluorescent protein) control adenovirus (young Ctrl), Sox9 overexpression adenovirus (Sox9 overexpression [OE]), and senescent VSMCs transduced with short hairpin EGFP (shEGFP) control adenovirus (senescent Ctrl) and Sox9 knockout adenovirus (Sox9 knockout [KO]; n=5 from 3 isolates). Normality was validated via the Shapiro-Wilk test and 2-way ANOVA with q-values adjusted for multiple testing with Benjamini-Hochberg false discovery rate (FDR) correction. Quantification and representative Western blot of LH3 protein expression in young Ctrl, Sox9 OE, senescent Ctrl, and Sox9 KO VSMC (**B** and **C**) cell lysate (n=5 from 3 isolates) and (**D** and **E**) ECM (n=3 individual isolates). Normality was validated via the Shapiro-Wilk test and 2-way ANOVA with q-values adjusted for multiple testing with Benjamini-Hochberg FDR correction. **F** and **G**, Quantification and representative immunofluorescence of fluorescent LH3 signal in the ECM synthesized from young Ctrl, Sox9 OE, senescent Ctrl, and Sox9 KO VSMCs (n=25 from 3 isolates). Normality was validated via the Shapiro-Wilk test and 2-way ANOVA with q-values adjusted for multiple testing with Benjamini-Hochberg FDR correction. **H**, Atomic force microscopy (AFM) stiffness measurements (kPa) in decellularized ECM synthesized from (**E**) young Ctrl (n=151), Sox9 OE (n=183), and Sox9 OE with *Plod3* knockout (*siPlod3*; n=197) and (**I**) senescent Ctrl (n=143), Sox9 KO (n=155), and *Plod3* knockout (senescent *siPlod3*; n=142). Measurements were taken from ECM synthesized from 3 isolates. The Shapiro-Wilk test for normality distribution was rejected; mixed model analysis with q-values adjusted for multiple testing with Benjamini-Hochberg FDR correction is shown.

We observed that Sox9 upregulated Col modifiers within the ECM that could potentially be responsible for the changes in ECM stiffness. *Plod3*/LH3, a protein with dual cross-linking and glycating properties, stood out as one of the few proteins, which was upregulated in the young Sox9 overexpression ECM (Figure 6). To validate the changes in LH3 in response to Sox9, we overexpressed and depleted Sox9 in young and senescent VSMCs and examined LH3 expression using RT-qPCR and Western blot. *Plod3* was upregulated in young Sox9 overexpressing VSMCs and downregulated in senescent Sox9 knockout VSMCs compared with the young and senescent controls, respectively (Figure 6A). On the protein level, LH3 was upregulated with Sox9 overexpression but not downregulated in Sox9 knockout compared with their respective controls (Figure 6B and 6C). Immunofluorescence showed that LH3 was consistently localized to the Golgi apparatus with no change in localization either with senescence or Sox9 expression (Figure S5A). However, examination of the decellularized ECM using immunofluorescence showed that LH3 deposition was markedly increased in the young Sox9 overexpression and senescent control conditions compared with the young control and senescent Sox9 knockout ECM where it was decreased (Figure 6D through 6G). LH3 also showed a distinctive pattern change with Sox9 overexpression, which mirrored the pattern seen in the senescent control ECM, adopting an increased fibrillar deposition pattern (Figure 6G). This suggested that increased deposition of LH3 into the matrix, rather than increased intracellular protein synthesis, may be involved in ECM modulation.

To test whether LH3 plays a role in Sox9-driven ECM stiffness, we depleted *Plod3* using RNAi in young Sox9 overexpressing and senescent control VSMCs (Figure S5B). Atomic force microscopy measurements revealed a marked reduction in ECM stiffness in matrices synthesized from young VSMCs treated with Sox9 overexpression adenovirus in combination with siPlod3 compared with the young Sox9 overexpression ECM. There was also no difference in stiffness compared with the young control ECM (Figure 6H). Knocking out *Plod3* in senescent VSMCs resulted in a reduction in ECM stiffness compared with the senescent control to a stiffness equivalent to that observed in Sox9 knockout ECM (Figure 6I). This demonstrated that LH3 may be a primary target driving Sox9–dependent ECM stiffness in aging.

**Figure 7.**
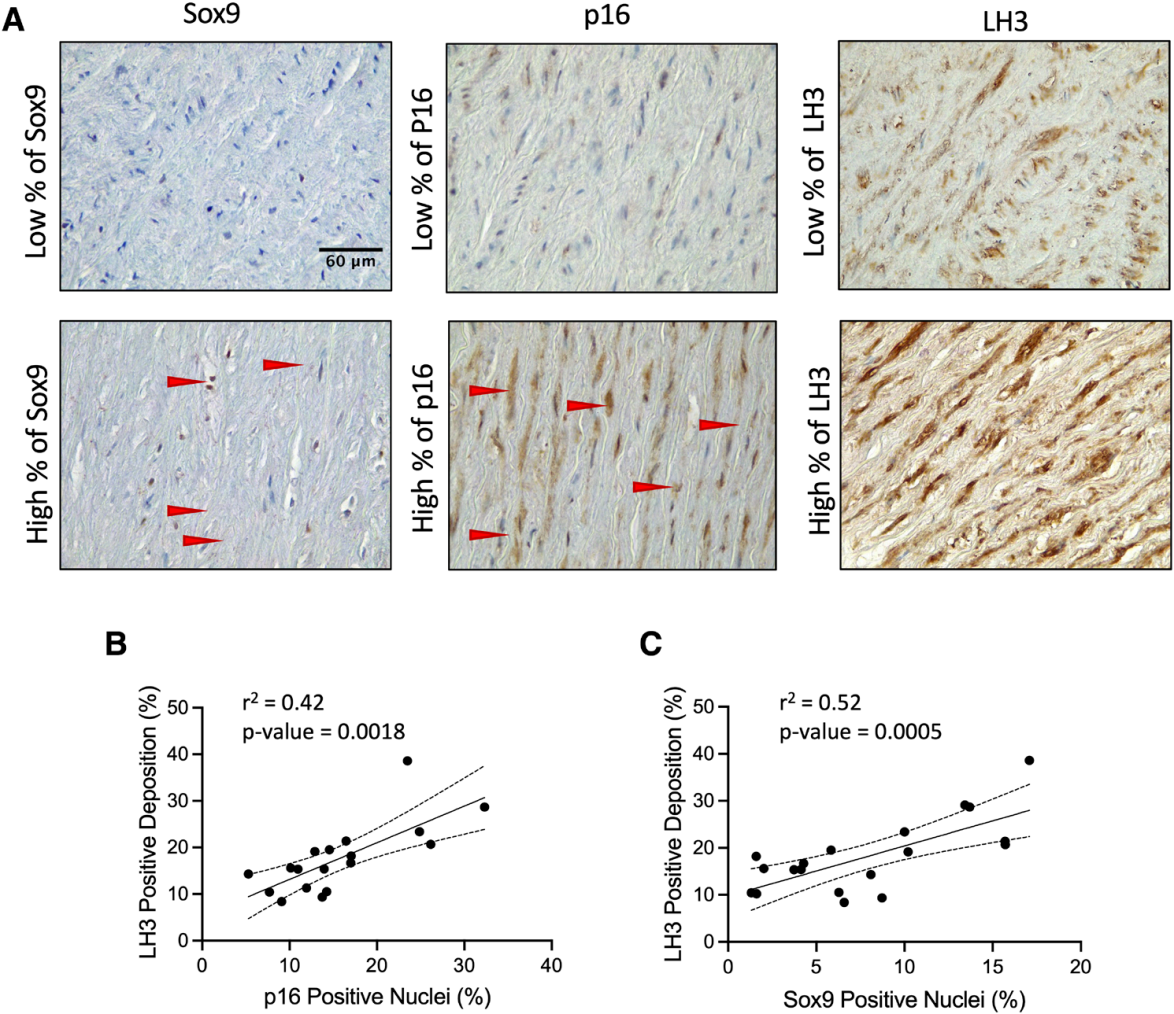
**LH3 (procollagen-lysine, 2-oxoglutarate 5-dioxygenase 3) deposition increases in the medial aortic layer with age and Sox9 (SRY-box transcription factor 9) expression. A**, Immunohistochemistry of Sox9, p16 (cyclin-dependent kinase inhibitor 2A), and LH3 staining in the aortic medial layer. Positive staining is shown in brown. Red arrows highlight the positive staining of Sox9 and p16. Correlation of LH3 positive staining (%) with (**B**) p16 and (**C**) Sox9 positive nuclei. Normality was validated via the Shapiro-Wilk test, and correlation was determined via the Pearson test (n=16).

To validate our findings in vivo, we again conducted immunohistochemistry on aortic patient tissue samples testing for LH3, Sox9, and p16 (Figure 7). We found that LH3 was increased in the aorta of aged individuals and that there was a correlation between elevated expression of LH3 and Sox9, as well as LH3 and p16, mirroring the previous correlations observed for Sox9 and p16 in patient tissue samples (Figure 7A through 7C).

### LH3 Deposition Into the ECM Is Regulated via EVs

**Figure 8.**
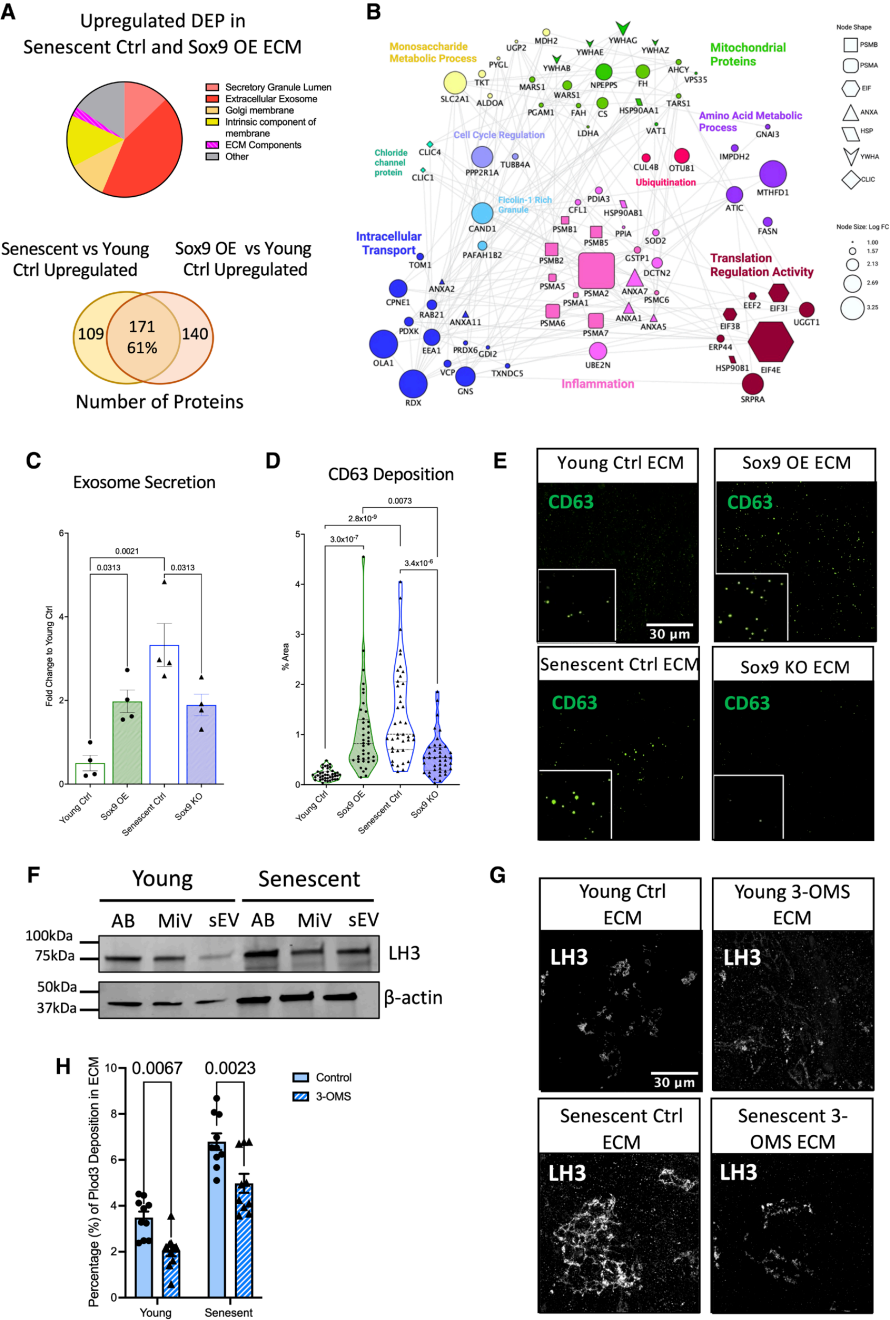
**Sox9 (SRY-box transcription factor 9) regulates LH3 (procollagen-lysine, 2-oxoglutarate 5-dioxygenase 3) deposition into the extracellular matrix (ECM) via increased extracellular vesicle (EV) secretion. A**, Pie chart and Venn diagram depicting categories and percentages of upregulated differentially expressed proteins (DEPs) found in the ECM synthesized from both senescent vascular smooth muscle cells (VSMCs) transduced with EGFP (enhanced green fluorescent protein; senescent Ctrl) and young VSMCs transduced with Sox9 overexpression (OE) adenovirus. **B**, Protein-protein interaction of upregulated DEP found in Sox9 OE ECM related to EVs through gene ontology. **C**, Quantification of exosome secretion in young VSMCs transduced with EGFP control adenovirus (young Ctrl), Sox9 OE, senescent Ctrl, and Sox9 knockout (KO) adenovirus (n=4 from 3 isolates). Normality was validated via the Shapiro-Wilk test and 1-way ANOVA with Tukey post hoc. **D** and **E**, Quantification (n=20 from 3 isolates) and representative immunofluorescence (IF) images of CD63 molecule deposition in the young Ctrl, Sox9 OE, senescent Ctrl, and Sox9 KO ECM. Normality was rejected via the Shapiro-Wilk test; mixed model analysis with q-values adjusted for multiple testing with Benjamini-Hochberg FDR correction is shown. **F**, Western blot of LH3 protein expression in EVs, apoptotic bodies (ABs), micro-vesicles (miVs), and small EV (sEV). **G** and **H**, Representative IF images and quantification (n=10 from 3 isolates) of LH3 deposition into young and senescent ECM with or without the sEV secretion inhibitor 3-O-methyl-sphingomyelin. Normality was accepted via the Shapiro-Wilk test; 2-way ANOVA with q-values adjusted for multiple testing with Benjamini-Hochberg FDR correction is shown.

EVs carry cargo from the cytosol that deposits within the extracellular space.^35^ The proteomics dataset showed that the upregulated proteins deposited in the ECM with Sox9 overexpression and senescence were related to EVs (Figure 8A). We again performed comparative analysis to determine what proteins were differentially expressed between senescent control and young Sox9 overexpression ECM when both were compared independently with the young control ECM. This showed that 171 proteins were in common representing a 61% overlap between the groups. Subclassifying these common upregulated proteins revealed that several were related to the regulation of intracellular transport, such as EEA1 (early endosome antigen 1) and Rab21 (Ras-related protein Rab21; Figure 8B). As LH3 has been shown to be trafficked into the ECM in Col IV carriers,^36^ we hypothesized that EVs may drive the increased deposition of LH3 into the ECM in VSMC senescence. To validate changes in EV secretion and deposition in response to Sox9 overexpression and senescence, we performed a CD63 bead capture assay and immunofluorescence for the EV marker CD63 to quantify exosome secretion (Figure 8C) and deposition into the ECM respectively (Figure 8D and 8E). These assays showed that Sox9 overexpression, as well as VSMC senescence, increased EV secretion and deposition, while Sox9 knockout resulted in the opposite effect. Using differential ultracentrifugation to isolate different EV populations, we showed that LH3 was detectable in all EV subtypes, including apoptotic bodies, micro-vesicles, and small vesicles in both young and senescent VSMCs (Figure 8F). However, no change in LH3 loading was found between the different EV subtypes or between young and senescent cells (Figure S6). To investigate the role of small vesicles in the secretion of LH3, we inhibited EV secretion using 3-O-methyl-sphingomyelin in both young and senescent VSMCs during ECM synthesis. We observed a reduction in LH3 deposition into the ECM supporting the notion that Sox9 drives increased release of EVs into the ECM during senescence, promoting enhanced deposition of LH3 and the modulation of ECM mechanics.

## DISCUSSION

This study explored the role and regulation of Sox9 in vascular aging. It reveals a crucial role for Sox9 in driving the senescent ECM phenotype of VSMCs by regulating ECM stiffness, fiber alignment, and composition. Our findings suggest that elevated expression of Sox9 in VSMCs strongly depends on mechanosignaling cues from the extracellular environment. We demonstrate that Sox9 regulates ECM composition by driving a phenotype closely mimicking the senescent VSMC ECM characterized by the downregulation of essential basement membrane proteins and the upregulation of LH3. Crucially, these Sox9-driven ECM changes can feedback and regulate the phenotype of VSMCs by modulating proliferation, inflammation, and senescence. This study also highlights the underappreciated role of LH3 as a driver of ECM stiffness and identifies EVs as novel regulators of ECM remodeling.

### Sox9 Is a key regulator of VSMC Phenotype During Aging

Our results have provided confirmation of previous studies that highlight the role of Sox9 in regulating VSMC contractility.^22,23^ We observed a negative correlation between Sox9 and α-SMA in vivo human aortic tissue samples, as well as in vitro via Sox9 overexpression. However, our results contrast with previous studies examining the interplay between Sox9 and vascular calcification.^23^ We found no correlation between medial calcification and Sox9 expression in vivo, and we verified this in vitro by showing no changes in calcification propensity between control and Sox9 overexpressing VSMCs. We propose these differences may be associated with the origins of the cells used. Previous studies finding an association between Sox9 and calcification were conducted in mouse models, which are genetically susceptible to spontaneous cartilaginous metaplasia that is prevalent in mice but rare in humans.^37^ Indeed, we found in human VSMCs that Sox9 did not increase the deposition of the chondrocyte markers *Collagen 2* and *Collagen 11* that have been colocalized with Sox9 in mouse aortic tissues.^23^ Runx2, a critical regulator of osteoblast differentiation^38^ and a driver of vascular calcification,^17,20^ has a tight interplay with Sox9, with both TFs exerting decreased transcriptional activation of one another.^39^ Thus, the VSMCs within the microenvironments where calcification occurs potentially have a dominant Runx2 expressing phenotype. As Sox9 promotes a stiff ECM niche that accelerates VSMC senescence, it may act as a priming factor for downstream calcification once its expression has diminished.^20^ These findings highlight the complexity of the interplay between VSMC dedifferentiation, the ECM and calcification, and the need for further investigation into the regulatory mechanisms underlying these processes.

We observed a correlation between Sox9 expression and p16, a marker for cellular senescence. In vitro studies showed that there was not a direct relationship between Sox9 levels and senescence; however, we found that Sox9 expression showed mechanosensitive responses, most strongly in senescent VSMCs, with increased expression and nuclear translocation observed on stiff surfaces. This observation is in line with previous studies, which have shown that Sox9 is mechanosensitive in chondrocytes, interestingly at the same 500-kPa stiffness that we observed in VSMCs, with this stiffness likely to be achieved in the aged vasculature.^30,40^ Although it is unclear whether the mechanosensitive responses of Sox9 are direct or downstream of other mechanosignaling pathways, our findings suggest that aortic stiffening, driven in part by the modified ECM deposited by senescent VSMCs, is conducive to increased expression of Sox9, which, in turn, leads to further elaboration of a senescent ECM.

### Sox9-Induced ECM Changes Influence VSMC Aging and Inflammation

We demonstrated the significant role of Sox9 in regulating ECM fiber alignment, ECM stiffness, and the phenotype of VSMCs. Our findings show that decreased cellular contractility, as a result of Sox9 expression, contributed to ECM fiber alignment, and we provide novel evidence that the Sox9-driven ECM compositional changes can feedback and regulate the phenotype of VSMCs. While previous studies have examined the role of ECM in cell signaling, stem cell differentiation,^41,42^ adhesion, and migration,^43,44^ the role of ECM in regenerating cells from a pathological state has remained understudied. Replating senescent cells on an ECM synthesized from Sox9-depleted VSMCs enabled, to some extent, the rejuvenation of cells with the reduction in senescence markers and reentry back into the cell cycle. Conversely, VSMCs plated on ECM from Sox9-expressing cells acquired features of cellular senescence including cell cycle arrest and DNA damage. We also showed the vital role of ECM alignment in VSMC orientation. Cells plated on fibrous ECMs, such as those synthesized from senescent and young Sox9-overexpressing VSMCs, were unable to form proper alignments with neighboring cells and fibers. Correct VSMC alignment is crucial in the vasculature, as it affects the mechanical properties of the tissue, influencing its ability to withstand and respond appropriately to mechanical stresses and strains.^45^ Our findings underscore the impact of Sox9-modulated ECM on the phenotype of VSMCs, and further work is now required to understand how complex compositional and structural changes contribute to VSMC aging and rejuvenation.

Our investigation also explored the compositional changes driven by Sox9 that increases ECM stiffness. Previous studies^25,26^ identified Sox9 as a potent activator of numerous ECM proteins in chondrocytes, leading us to anticipate an increase in Col deposition. However, we found that increased Sox9 expression in VSMCs resulted in the downregulation of a wide range of Cols and glycoproteins. This observation highlights previous research showing that Sox9 can serve both as an activator and a repressor,^46^ as well as a regulator of both ubiquitous and chondrocyte-specific genes.^47^ Our findings suggest that the transcriptional targets of Sox9 vary significantly depending on the cell type in which it is expressed. In VSMCs, Sox9 acts as a repressor for the transcription of various basement membrane proteins, whose decreased expression can negatively impact both the phenotype of VSMCs^48^ and the tensile strength of the vessel wall.^49^ Indeed, we validated both increased LH3 expression and decreased Col IV. LH3 is essential for normal biosynthesis and secretion of type IV Cols^50^ into the basement membrane, and both these proteins have been implicated in vascular diseases, including Ehlers-Danlos syndrome, which is associated with aneurysm and vessel rupture (Online Mendelian Inheritance in Man [OMIM] 612394).^49^ Our study suggests that loss of basement membrane integrity may also be a key feature of vascular aging, increasing the susceptibility of the vessel to structural failure. Loss of basement membrane proteins may also impact endothelial-mesenchymal transition, a key pathway in vascular remodeling.^51^

### LH3 and EVs as Key Modulators of ECM Stiffening

LH3 plays an important role in Col cross-linking and glycation both intracellularly ^52^ and extracellularly.^50^ Our results demonstrated that Sox9 and cellular senescence increased LH3 deposition in the ECM, and this was confirmed in vivo where LH3 correlated with Sox9 and p16 expression in aged aortic samples. In vitro, we showed that Sox9-regulated ECM stiffness was primarily driven by LH3, as its depletion resulted in a reduction of ECM stiffness, similar to that observed with the depletion of Sox9. Previous research has shown that LH3 is trafficked into the extracellular space via Col IV carriers,^36^ and our analysis of EV secretion from young and senescent VSMCs revealed that LH3 is loaded onto all EV types. These findings suggest that the increased deposition of LH3 in the ECM is due to both Sox9-mediated intracellular expression and increased secretion of EVs. Importantly, these carriers can signal remotely from the cell, suggesting that a small number of VSMCs releasing these particles may have profound effects on ECM stiffening in the local environment. This highlights LH3 and EV secretion as potential therapeutic targets for further investigation in cardiovascular pathologies.

## ARTICLE INFORMATION

### Acknowledgments

The proteomics was performed at the Denmark Hill Proteomics Facility. Graphical abstract and schematic were created with BioRender.com. The microscopy was performed at the Wohl Cellular Imaging Centre, King’s College London.

### Author Contributions

M. Faleeva, S. Cox, and C.M. Shanahan contributed to conception; M. Faleeva, M. Whitehead, E. Marhuenda, T. Iskratsch, S. Cox, and C.M. Shanahan to experimental design; M. Faleeva, S. Ahmad, and G. Watson to acquisition of data; and M. Faleeva, S. Ahmad, and K. Theofilatos to analysis and interpretation of data. M. Faleeva and C.M. Shanahan wrote and revised the article. All authors provided final approval of the submitted version.

### Sources of Funding

This study was supported by the British Heart Foundation (BHF) Programme grant RG/F/21/110064 to C.M. Shanahan and the King’s College London BHF Centre of Research Excellence Interdisciplinary PhD studentship to S. Cox and C.M. Shanahan.

### Disclosures

None.

### Supplemental Material

Expanded Materials and Methods

Table S1

Figures S1–S6

References 53–65

## Supplementary Material


