# Supplementary material for: Sox9 Accelerates Vascular Aging by Regulating Extracellular Matrix Composition and Stiffness
Source: Circ Res. 2024 Jan 5;134(3):307–24. doi: 10.1161/CIRCRESAHA.123.323365 (PMC10826924; doi:10.1161/CIRCRESAHA.123.323365)
Supplement: Supplementary file 1 [file res-134-307-s001.pdf]

Major Resources Table

Antibodies

Target antigen	Vendor or Source	Catalog #	Working concentration	Lot # (preferred but not required)	Persistent ID / URL
Sox9	Abcam	ab185966	1:200 (IHC)<br>1:1000 (WB)<br>1:100 (IF)		https://www.abcam.com/products/primary-antibodies/sox9-antibody-epr14335-78-ab185966.html
P16	Abcam	ab241543	1:500 (IHC)		https://www.abcam.com/products/primary-antibodies/cdkn2ap16ink4a-antibody-pablo33b-ab241543.html
α-SMA	Sigma Aldrich	A5691	1:50 (IHC)		https://www.sigmaaldrich.com/GB/en/product/sigma/a5691
CD68	Santa Cruz Biotechnology	Sc-70761	1:500 (IHC)		https://www.scbt.com/p/cd68-antibody-3f103
Plod3	Proteintech	11027-1AP	1:100 (IHC)<br>1:1000 (WB)<br>1:250 (IF)		https://www.ptglab.com/products/PLOD3-Antibody-11027-1-AP.htm
α-SMA	Abcam	ab7818	1: :5000 (WB)		https://www.abcam.com/products/primary-antibodies/alpha-smooth-muscle-actin-antibody-1a4-ab7817.html
GAPDH	Sigma Aldrich	MA1-16757	1:5000 (WB)		https://www.thermofisher.com/antibody/product/GAPDH-Antibody-clone-1D4-Monoclonal/MA1-16757
Beta actin	Sigma Aldrich	A1978	1:5000 (WB)		https://www.sigmaaldrich.com/GB/en/product/sigma/a1978
IRDye 800CW Donkey anti-Rabbit	LI-COR Biosciences	926-32213	1:10000 (WB)		https://www.licor.com/bio/reagents/irdye-800cw-donkey-anti-rabbit-igg-secondary-antibody
IRDye 680RD Donkey anti-Mouse	LI-COR Biosciences	926-68072	1:10000 (WB)		https://www.licor.com/bio/reagents/irdye-680rd-donkey-anti-mouse-igg-secondary-antibody
Fibronectin	Abcam	ab2413	1:500 (IF)		https://www.abcam.com/products/primary-antibodies/fibronectin-antibody-ab2413.html
CD63	Santa Cruz	s-15,363	1:200 (IF)		https://www.scbt.com/p/cd63-antibody-h-193

DOI [to be added]

Vinculin	Sigma Aldrich	V9264	1:500 (IF)		https://www.sigmaaldrich.com/GB/en/product/sigma/v9264
gamma-H2AX	Cell Signaling	2577	1:200 (IF)		https://www.cellsignal.com/products/primary-antibodies/phospho-histone-h2a-x-ser139-antibody/2577?_requestid=2679268
Alexa Fluor 546 donkey anti-mouse IgG	Invitrogen	A10036	1:400 (IF)		https://www.thermofisher.com/antibody/product/Donkey-anti-Mouse-IgG-H-L-Highly-Cross-Adsorbed-Secondary-Antibody-Polyclonal/A10036
Alexa Fluor 488 goat anti-rabbit IgG	Invitrogen	A11008	1:400 (IF)		https://www.thermofisher.com/antibody/product/Goat-anti-Rabbit-IgG-H-L-Cross-Adsorbed-Secondary-Antibody-Polyclonal/A-11008
Phalloidin	Invitrogen	A30107	1:400		https://www.thermofisher.com/order/catalog/product/A30107
DAPI	Sigma Aldrich	D9542	1:10000		https://www.sigmaaldrich.com/GB/en/product/sigma/d9542
Cd63	BD Biosciences	556019	35µg		https://www.bdbiosciences.com/en-gb/products/reagents/flow-cytometry-reagents/research-reagents/single-color-antibodies-ruo/purified-mouse-anti-human-cd63.556019
PE Mouse Anti-human CD81	BD Pharmigen	555676	1:50		https://www.citeab.com/antibodies/2412825-555676-bd-pharmingen-pe-mouse-anti-human-cd81

DNA/cDNA Clones

Clone Name	Sequence	Source / Repository	Persistent ID / URL
N/A			

Cultured Cells

Name	Vendor or Source	Sex (F, M, or unknown)	Persistent ID / URL
04.35F.11A	Primary cells	F	
03.38F.11A	Primary cells	F	
05.33F.5A	Primary cells	F	

Other

Description	Source / Repository	Persistent ID / URL
Sodium Citrate Buffer	Vector Laboratories, H-3300	https://vectorlabs.com/products/antigen-unmasking-solution-citric-acid-basedproduct-info-overview/

DOI [to be added]

Avidin-biotin complex (ABC) Reaction	Vector Labs kits PK-6102 & PK-6010	https://vectorlabs.com/products/vectastain-elite-abc-hrp-kit-mouse-igg/
3, 3'-diaminobenzidine (DAB) peroxidase substrate kit	Vector Laboratories; SK-4100	https://vectorlabs.com/products/dab-hrp-substrate/
RNA STAT-60	Tel-Test Inc, CS-111	https://www.amsbio.com/rna-stat-60-cs-111
DEPC treated H_2O	Ambion AM9915G	https://www.thermofisher.com/order/catalog/product/AM9915G
GoScript™ Reverse Transcriptase	Promega A5001	https://www.promega.co.uk/products/pcr/rt-pcr/goscript-reverse-transcriptase/?catNum=A5003
SYBR Green Master Mix	PCR Bio, PB012619-120-22	https://pcrbio.com/products/pcr/pcrbio-taq-dna-polymerase-mixes/
sulfa SANPAH solution	Thermo scientific, 22589	https://www.thermofisher.com/order/catalog/product/22589
Collagen 1	Gibco A10483	https://www.thermofisher.com/order/catalog/product/A1048301
Click-iT Edu	ThermoFisher, C10337	https://www.thermofisher.com/order/catalog/product/C10337
aldehyde-sulphate beads	Invitrogen, A37304	https://www.thermofisher.com/order/catalog/product/A37304
MES buffer	Sigma, 76039	https://www.sigmaaldrich.com/GB/en/product/sigma/76039
Solution13	ChemoMetec, 910-3013	https://chemometec.com/reagents/solution-13/?gad=1&gclid=CjwKCAjwsKqoBhBPEiwALrqiIBqzFju0B6JHFKh-e0dVBW7cAasK861AF4QO5kZlCuP9b4BORa6-xoCS3YQAvD_BwE
Chondroitinase ABC from Poteus vulgaris	Sigma, C3667-5UN	https://www.sigmaaldrich.com/GB/en/product/sigma/c2905
Heparinase II from Flavobacterium heparinum	Sigma, H6512-10UN	https://www.sigmaaldrich.com/GB/en/product/sigma/h6512
Endo-beta-galactosidase from Bacteroides fragilis (Keratanase)	Sigma, G6920-.5UN	https://www.sigmaaldrich.com/GB/en/product/sigma/g6920
Glycoprotein Deglycosylation kit (3 de-branching enzymes + 1 O-deglycosidase)	EMD Millipore, 362280	https://www.sigmaaldrich.com/GB/en/product/mm/362280
N-Glycosidase F (PNGaseF)	Sigma Aldrich, 362185-100U	https://www.sigmaaldrich.com/GB/en/product/mm/362185
4–20% precast polyacrylamide gel	Biorad, 4561096	https://www.bio-rad.com/en-uk/sku/4561096-4-20-mini-protean-tgx-precast-protein-gels-15-well-15-ul?ID=4561096

DOI [to be added]

HiPerfect Transfection Reagent	Qiagen, 301707	https://www.qiagen.com/us/products/discovery-and-translational-research/functional-and-cell-analysis/transfection/hiperfect-transfection-reagent
Fluoromount-G	Invitrogen, 00-4958-02	https://www.thermofisher.com/order/catalog/product/00-4958-02
Dulbecco's modified eagle medium (DMEM)	Sigma, D5921	https://www.sigmaaldrich.com/GB/en/product/sigma/d5921

DOI [to be added]
