# Supplementary material for: Sox9 Accelerates Vascular Aging by Regulating Extracellular Matrix Composition and Stiffness
Source: Circ Res. 2024 Jan 5;134(3):307–24. doi: 10.1161/CIRCRESAHA.123.323365 (PMC10826924; doi:10.1161/CIRCRESAHA.123.323365)

Figure 1c: Sox9 expression in young and senescent VSMCs

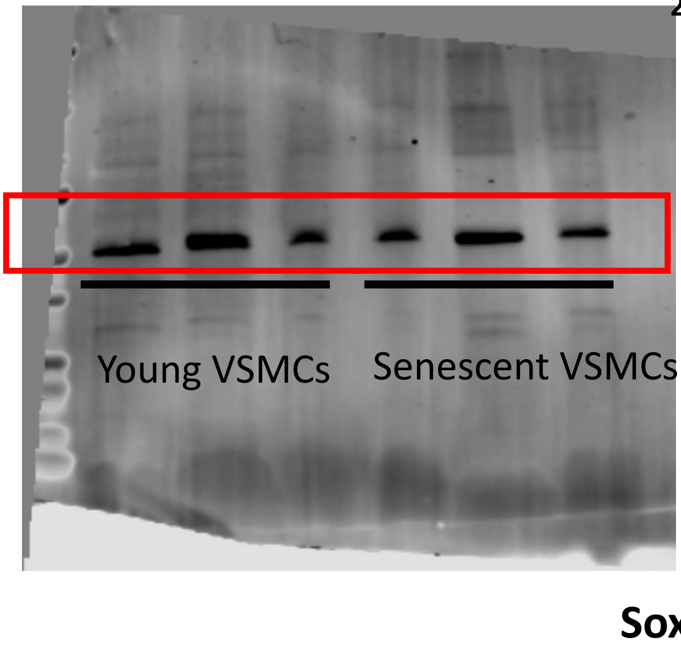

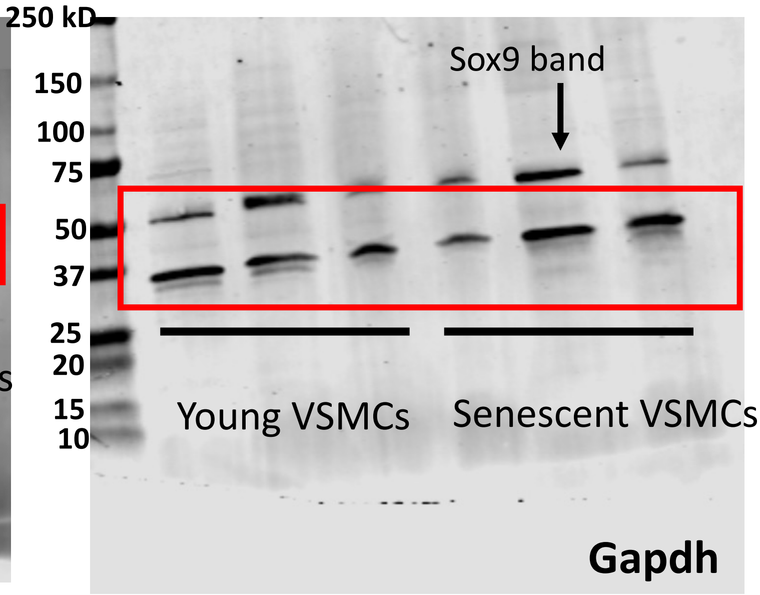

Figure 2j: Sox9 expression on hydrogels

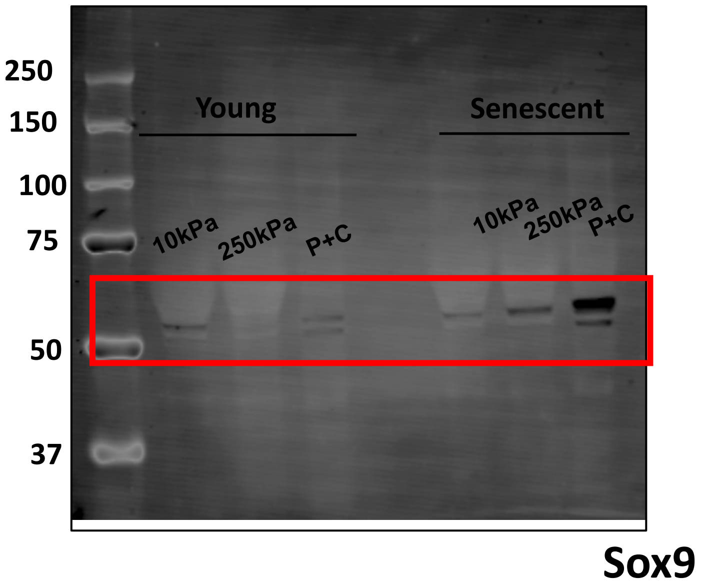

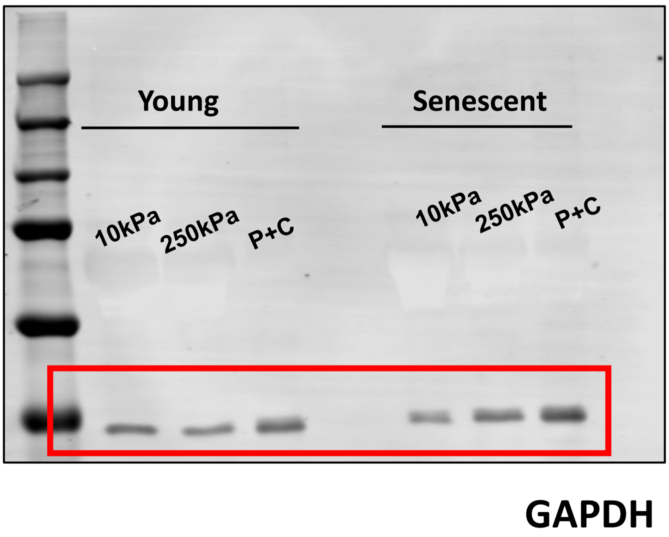

Put ladder sizes and mark the protein band

Figure 6c: LH3 in Cell Lysate

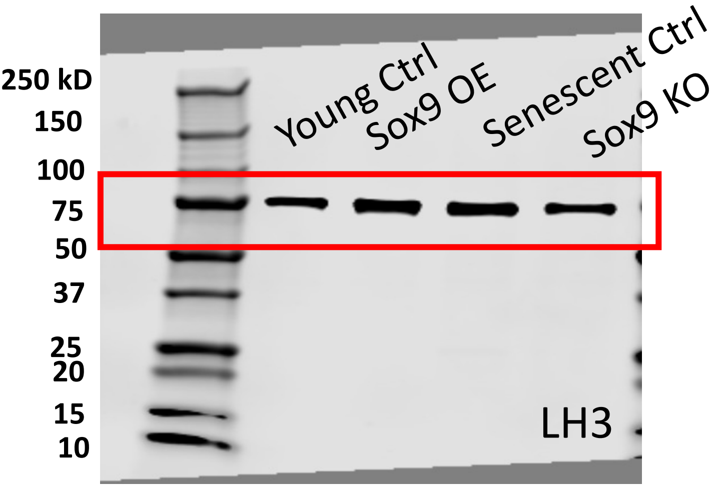

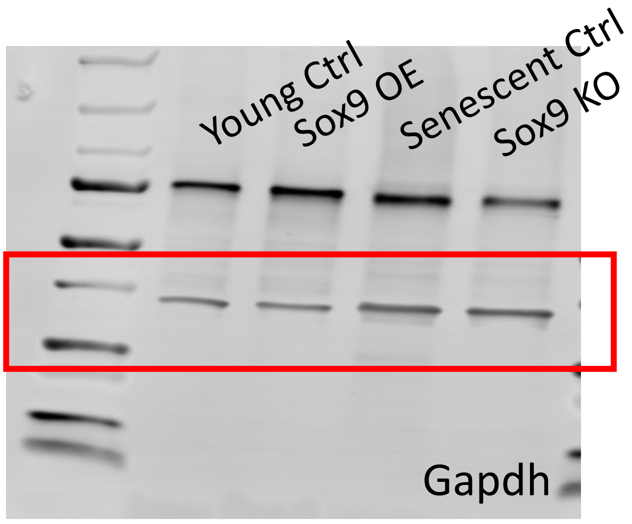

Figure 6d: LH3 in ECM Lysate

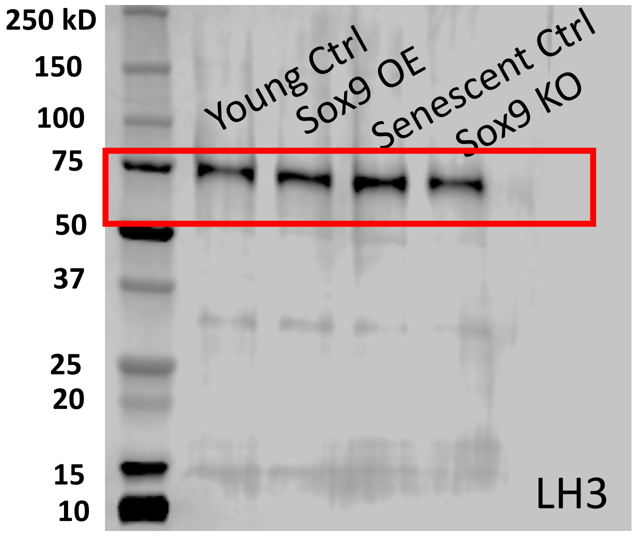

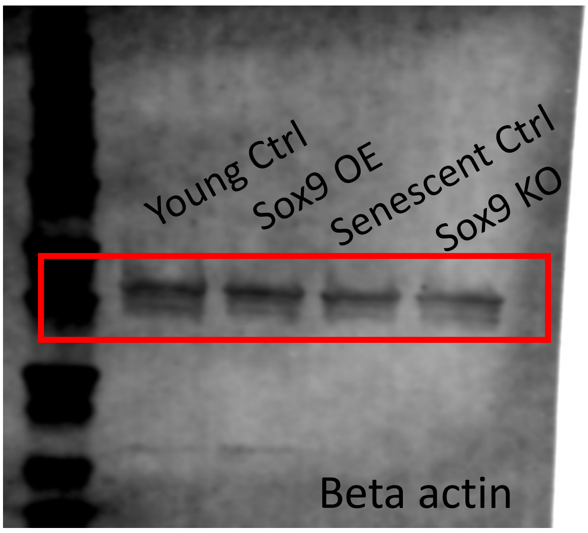

Figure 8f: LH3 in Extracellular Vesicles

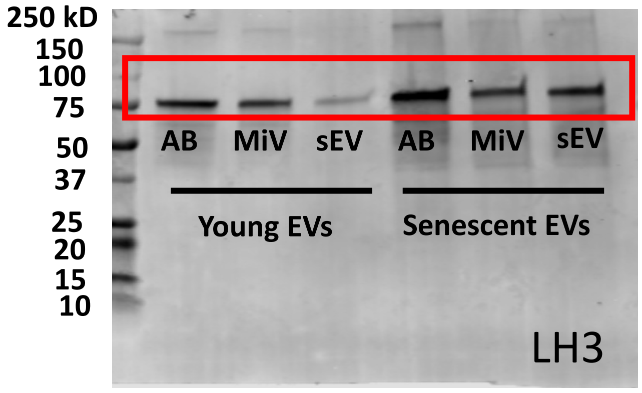

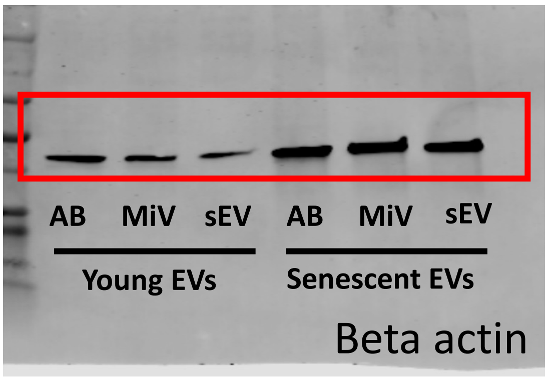

Supplementary Figure 2c:

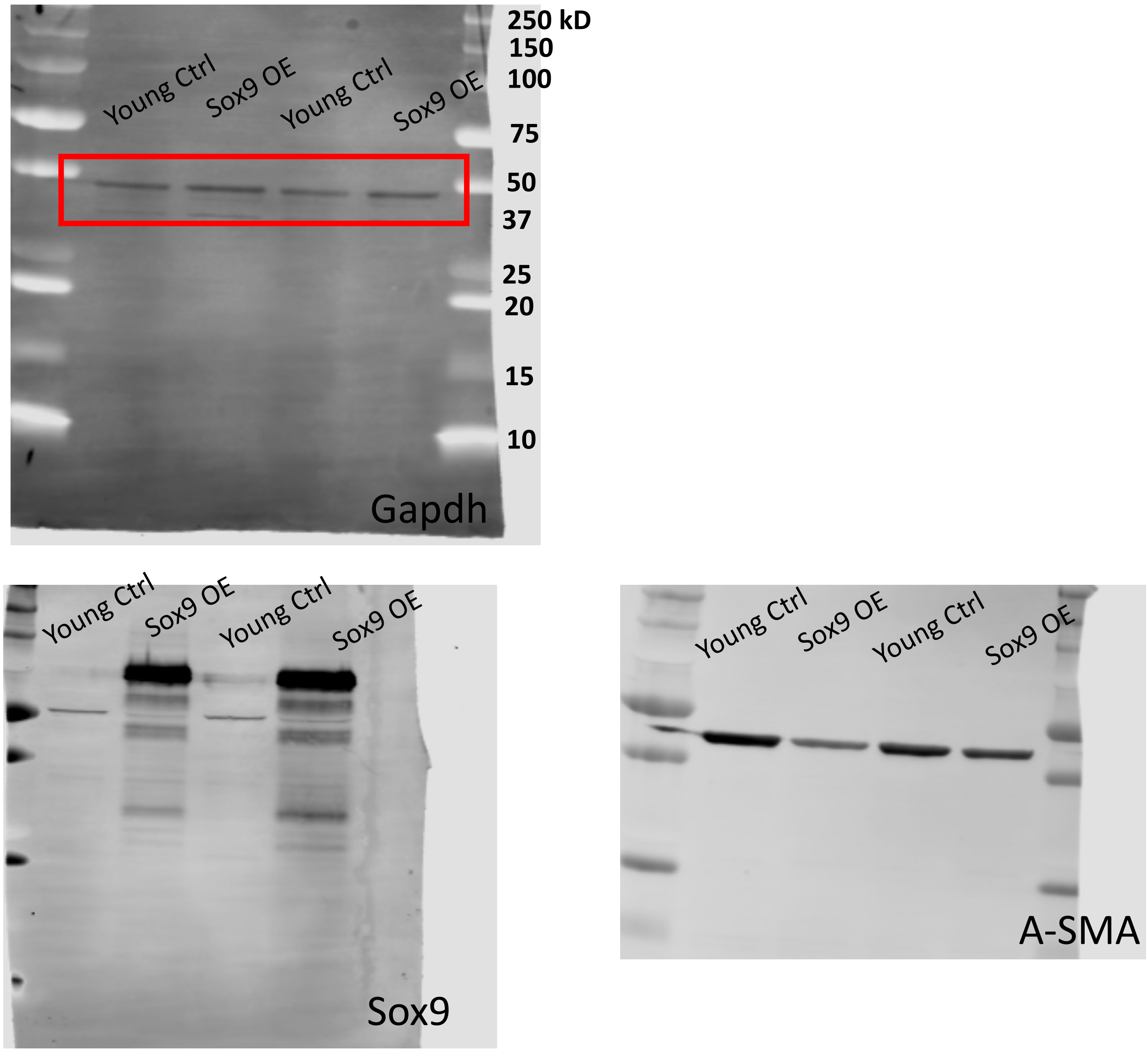

Supplementary Figure 2d:

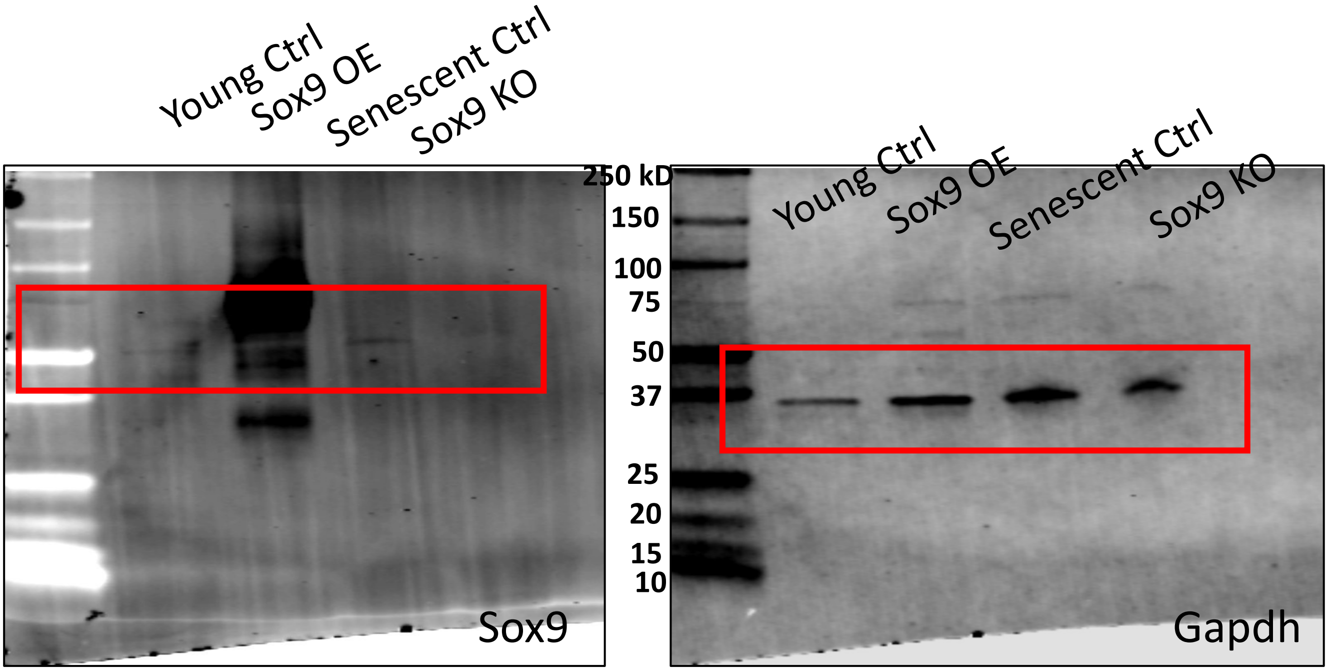

Supplementary Figure 5b:

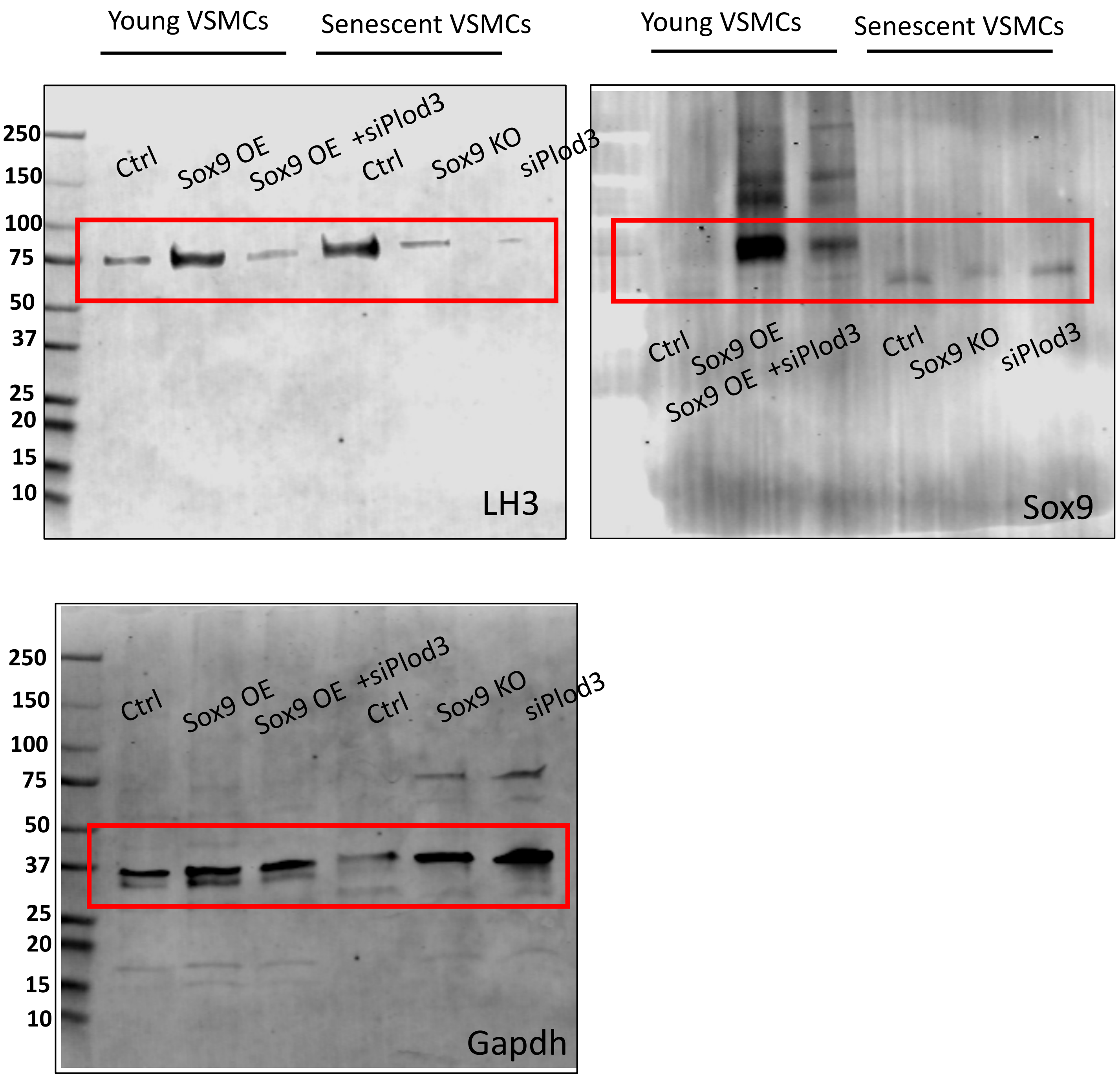

Supplement: Supplementary file 2 [file res-134-307-s002.pdf]
