# Supplementary material for: Sox9 Accelerates Vascular Aging by Regulating Extracellular Matrix Composition and Stiffness
Source: Circ Res. 2024 Jan 5;134(3):307–24. doi: 10.1161/CIRCRESAHA.123.323365 (PMC10826924; doi:10.1161/CIRCRESAHA.123.323365)
Supplement: Supplementary file 3 [file res-134-307-s003.pdf]

# Supplemental Materials

# Sox9 accelerates vascular ageing by regulating extracellular matrix composition and stiffness.

Maria Faleeva[1], Sadia Ahmad[1], Konstantinos Theofilatos[1], Steven Lynham[2,] Gabriel Watson[1], Meredith Whitehead[1], Emilie Marhuenda[3], Thomas Iskratsch[3], Susan Cox[3], Catherine M Shanahan[1*].

[1] BHF Centre of Research Excellence, School of Cardiovascular and Metabolic Medicine & Sciences, King's College London, London, UK
[2] Proteomics Facility, Centre of Excellence for Mass Spectrometry, King's College London, UK
[3] School of Engineering and Material Science, Queen Mary University of London, London, UK
[4] Randall Centre for Cell & Molecular Biophysics, Faculty of Life Sciences & Medicine, King's College London, London, UK

**Corresponding Author:**
Professor Catherine M. Shanahan
School of Cardiovascular and Metabolic Medicine & Sciences,
King's College London,
London,
SE5 9NU
UK
Email: cathy.shanahan@kcl.ac.uk
Phone: +44 2078485221

**Running Title: Sox9 regulates vascular extracellular matrix ageing.**

**Supplemental Materials**

**Expanded Materials and Methods:**

**Immunohistochemistry:**

All human materials were handled in compliance with the Human Tissue Act (2004, UK) with informed consent and ethical approval from the National Research Ethics Service (REC reference: 13/LO/1950). Human aortic tissue samples used are shown on Supplementary Table 1. Immunohistochemistry was performed on 7um thick paraffin-embedded human aortic tissue sections. These sections were deparaffinized and rehydrated prior to heat-mediated antigen retrieval in sodium citrate buffer (Vector Laboratories, H-3300). Endogenous peroxidase activity was quenched by 3% H2O2, in methanol and non-specific binding blocked with 10% serum from the source species for the secondary antibody followed by a primary antibody incubation overnight at 4 °C (Sox9, abcam, ab185966,1:200); p16, (abcam, ab241543, 1:500), α-SMA ( Sigma-Aldrich, A5691, 1:50), CD68 (Santa Cruz Biotechnology, sc-70761, 1:500), LH3 (Proteintech, 11027-1AP, 1:100). Biotinylated secondary antibody was added, followed by avidin-biotin complex (ABC) reaction (Vector Labs kits PK-6102 & PK-6010). Staining was developed with 3, 3'-diaminobenzidine (DAB) peroxidase substrate kit (Vector Laboratories; SK-4100) and these sections were counterstained with Hematoxylin. The slides were mounted with DPX followed by dehydration and images were taken using a Leica ICC50 W Microscope, a minimum of eight images were taken of the tissue sample per aortic region (intima, media, adventitia). Signal optimisation of primary antibodies was performed by testing multiple concentrations and target staining was verified using secondary antibody only controls.α-SMA and CD68, a marker of macrophage infiltration, were used to delineate the cell types present in the samples. Percentage of positively stained nuclei was quantified using ImageJ software, with brown nuclei regarded as positive and blue/purple as negative. Percentage of positive extracellular staining was quantified via the threshold detection method on ImageJ.

**Cell culture:**

Human VSMCs were obtained by explant culture of thoracic aortic tissue obtained from healthy donors as previously described[30]. Briefly, 2mm pieces of tissue were cut from the deep medial layer and placed in a six well plate with M199 media (Sigma M2154), supplemented with 20% fetal bovine serum (FBS). A coverslip was placed over the tissue and when explanted cell growth had reached confluence cells were trypsinised (Sigma, T3924) for passaging. At first trypsinisation VSMCs were plated onto coverslips and immunofluorescence for smooth muscle markers SM-actin and calponin was used to establish cell population purity. Three different healthy donor isolates were used for this study; 35-year old female (04.35F.11A), 38-year old female (03.38F.11A), and 33-year old female (05.33F.5A). Cells were cultured at 37 °C and 5% $CO_2$ in M199 media supplemented with 20% FBS and 1% Penicillin-Streptomycin-Glutamine (PSG) (100x). Replicative senescence was induced by serial passaging of the cells at a ratio of 1:2 until growth arrest, typically occurring between passages 20-25. All experiments were performed using these 3 biological replicates, with multiple technical replicates taken from the three isolates at a different passage from originally used. Early passage VSMCs were collected at passages 6-13 and were termed 'young VSMCs' and viewed as a model of healthy cells as they showed robust proliferation and no evidence of DNA damage. Late passage cells, which have undergone multiple passages and reached a state of replicative senescence (termed 'senescent VSMCs'), were characterized by cessation of proliferation, altered morphology, positivity for senescence associated beta-galactosidase, elevated levels of DNA damage and expression of the cell cycle regulators p16 and p21 as previously described[31].

**Extracellular matrix synthesis:**

Decellularized ECM was synthesized as previously described [32]. Plates or cover slides were coated with 0.2% filtered gelatin solution at 37°C for 1 hour, cross-linked with 1% glutaraldehyde for 30 minutes at room temperature, and quenched with 1M ethanolamine for another 30 minutes at room temperature. Cells were seeded on the gelatin-coated plates at

high confluency and cultured in 20% FBS, 1% PSG M199 media supplemented with 50ug/mL L-ascorbate for 9-12 days. The ECM was decellularized by incubating the cells for 5 minutes with the Extraction Buffer (0.1% Triton, 20mM $NH_4OH$, diluted in PBS), then washed three times with PBS to remove cell debris.

**Adenoviral Constructs and Transfections:**

Sox9 was expressed in young VSMCs via a recombinant adenoviral Enhanced Green Fluorescent Protein (EGFP)-CMV-Sox9 (VectorBuilder) construct using $1.7 \times 10^7$ Infectious units per ml IFU/mL) and knocked out in senescent VSMCs via a recombinant EGFP-SOX9 shRNA (short hairpin RNA against Sox9) adenovirus (Vectorbuilder) (GCATCCTTCAATTTCTGTATACTCGAGTATACAGAAATTGAAGGATGC) using $4.2 \times 10^6$ IFU/mL. Control young and senescent VSMCs were treated with either EGFP (VectorBuilder) or Scramble shRNA control adenovirus (VectorBuilder) (CCTAAGGTTAAGTCGCCCTCG) using $1.7 \times 10^7$ IFU/mL for both. To determine the optimal viral titre that resulted in high transduction efficiency but did not impact on cell heath the virus was serially diluted. Cells were transduced in serum free M199 media containing the adenovirus for 40 minutes, and then diluted in M199 media with 20% FBS and 0.1% PSG for three days. Cells synthesising matrices were re-transduced every 5 days with L-ascorbate supplementation for a total of 9 days. 100% transduction efficiency was assessed via EGFP fluorescence imaging.

**siRNA Mediated Protein Depletion:**

Young and senescent VSMCs previously exposed to either Sox9 overexpression (OE) or shEGFP adenoviral treatments, underwent additional siRNA treatments aimed at depleting Plod3 (On-TARGETplus Human Plod3 siRNA, Dharmacon). The siRNA mastermix was prepared by combining 1 mL of serum-free media with 3 µL of a 20 µM siRNA solution and 60 µL of HiPerfect Transfection Reagent (Qiagen, Catalog #301707). This mastermix was briefly vortexed to ensure proper mixing and subsequently incubated at room temperature

for 10 minutes. Following mastermix preparation, the cell culture media was switched to M199 supplemented with 5% FBS and PSG. The siRNA mastermix was then applied to the cells, with 200 µL added per 6-well plate and 100 µL per 24-well plate. Subsequently, the culture media was refreshed after a 3-day incubation period, and the cells were returned for a further 2-day incubation in media supplemented with 20% FBS.

**Calcification Assays:**

VSMCs were treated with 5% FBS supplemented M199 media containing 2.7mmol/L Calcium and 2.5mmol/L Phosphate for 8 days, when signs of calcification became apparent. Control medium was 5% FBS supplemented M199 media without added calcium and phosphate. Calcification assays were conducted as previously described [20].

**RNA Extraction, Reverse Transcription, and qPCR:**

RNA from cultured VSMCs was isolated using RNA STAT-60 phenol-chloroform (Tel-Test Inc, CS-111). cDNA was synthesized using Mu-MLV reverse transcriptase (Promega, M1701) with dNTPS, random and oligo primers, and RNAse inhibitor (Promega A5001). qPCR (quantitative PCR) was performed using qPCRBIO SyGreen Mix (PCR Bio, PB012619-120-22) and run in a StepOnePlus Real Time PCR system. PCRs were performed in 20uL reaction volumes, with the thermocycler program running 95 °C for 10 minutes followed by 40 cycles at 95 °C for 5 seconds and 60 °C for 30 seconds. The following primers used; 18S (QuantiTec Primer Hs-RRN18s), *Sox9* (Sox9, 5’-AGCTCTGGAGACTTCTGAACGAGA -3’, and 5’ – ACTTGTAATCCGGGTGGTCCTTCT -3’, 75bp), *ACTA2* (5’- TGACAATGGCTCTGG GCTCTGTAA -3’, and 5’-TTCGTCACCCACGTAGCTGTCTTT -3’, 118bp), *TAGLN* (5’- TTG AAGGCAAAG ACATGGGAGCAG -3’, and 5’- TCCACGGTAGTGCCCATCATTCTT -3’, 88bp), *CDN2A* (QuantiTec Primer Hs- CDKN2A_vb.1_SG ), *CDKN1A* (QuantiTec Primer Hs-CDKN1A_1_SG ), *IL6* (QuantiTec Primer Hs-IL6_SG ), *IL8* (QuantiTec Primer Hs-CXCL8_1_SG ), *PLOD3* (PLOD3, 5’- TCTACACACGGCTCTACC-3’ and 5’-

ACCACTTCATCTAAAGCCC-3’, 91bp), *COL4A1* (COL4A1, 5’-GGCAGCCAGGACCTAAA-3’, and 5’-CCCATTCCACCAACAGAT-3’, 74bp), *COL4A2* (COL4A2, 5’-ACAAGTCCTACTGGCTCTCTA-3’, and 5’-GGCCTCACACACAGAAC-3’, 80bp), *NID2 (*NID2, 5’- TTCCTTCTCCTGCCGTTGTC-3’, and 5’- TGCTGTTGTTCACAGGGTGT-3’,76bp), Runx2 (QT00020517 Hs_RUNX2_1_SG QuantiTect Primer), BMP2 (QT00012544 Hs_BMP2_1_SG QuantiTect Primer), MSX2 (MSX2, 5’-AAATTCAGAAGATGGAGCGGCGTG-3’, and 5’- CGGCTTCCGATTGGTCTTGTGTTT-3’, 120bp). Expression levels of target genes were normalised to 18S expression values in young VSMCs using the 2-ΔΔCt method. Ct values were normalized to those of young or senescent cells plated on plastic in hydrogel and ECM cell re-plating experiments.

**Protein Extraction and Western blotting:**

ECM and cell lysate was homogenized in RIPA buffer with protease inhibitor cocktail, sonicated on ice, and centrifuged for 10 minutes at 7,500 at 4°C. 50% glycerol, 0.1% SDS, 30% 1M Tris Buffer pH=6.9, Bromophenol blue 6 mM, and 10% 10mM dithiothreitol (DTT) was added to the samples prior to boiling them for 10 minutes at 95°C. Proteins were separated via gradiet 4–20% precast polyacrylamide gel (Biorad, 451096) SDS-PAGE, transferred to Polyvinylidine Fluoride (PVDF) membrane, and blotted with 5% BSA in PBS-Tween (0.1%) (PBS-T) for one hour at room temperature. Membranes were incubated overnight at 4°C with the primary antibodies: Sox9 (Abcam, ab185966, 1:1000), A-SMA (Abcam, ab7818, 1:5000), LH3 (Proteintech, 11027-1AP, 1:1000). IRDye 800CW conjugated secondary antibodies (LI-COR Biosciences) were incubated for one hour at room temperature and visualized using an images (Odyssey; LI-COR Biosciences). Protein bands were quantified through ImageJ, normalized to either GAPDH or beta-actin. Actual size of the resolved Molecular weight (MW) standards are provided for each immunoblot.

**Hydrogel and Polydimethylsiloxane (PDMS) Setup:**

Hydrogel recipes measuring 10kPa and 250kPa were made up from 10.75% and 15% acrylamide and 9% and 1.2% bisacrylamide, respectively and stiffness was confirmed using AFM. Glass coverslips were coated with 3-aminopropyl trimethoxysilane (97%) for 2 minutes, washed thoroughly, and fixed for 30 minutes at room temperature with 1% glutaraldehyde with $ddH_2O$. Coverslips were dried face up overnight. Per each volume of acrylamide solution, 1/100$^{th}$ of the volume of APS (10%) and 1/1000$^{th}$ of the volume of TEMED was added. The solution was mixed thoroughly and aliquoted out onto glass slides. The fixed coverslips were placed onto the hydrogel solution facedown. The hydrogel solution was allowed to solidify and adhere onto the glass coverslips. The glass coverslips were then inverted, hydrogel slide up, and subjected to ultraviolet light (UV) for 5 minutes with a 2% of sulfa SANPAH solution (Thermo scientific, 22589). The hydrogels were washed several times under sterile conditions and coated with 3.3% of Rat Tail Collagen 1 (Gibco A10483) solution at 4°C for 2 hours. The collagen solution was then removed and cells were plated onto the hydrogels as normal.

Polydimethylsiloxane (PDMS) substrates were prepared as described previously [33] by mixing thoroughly the PDMS Sylgard 527 with Sylgard 194 with the ratio 1;0 (purre Sylgard 527), 1:20, 1:10, 1:8, 1:5 and 1:2 to obtain the stiffness 1,6, 20,60, 130, and 500kPa respectively. After being spin-coated onto coverslips, the PDMS was cured either at 80 °C for 2h or 60 °C overnight. PDMS was coated with 0.2% gelatin for 30 minutes at room temperature. Stiffness was measured using rheology as described previously[33].

**Immunofluorescence:**

VSMCs and ECM were fixed in 4% paraformaldehyde in PBS, and permeabilized with 0.5% PF-40. Permeabilization step was omitted in case of ECM staining. Cells were then blocked at room temperature with 3% BSA in PBS, and incubated with the primary antibodies; Fibronectin (ab2413, 1;500), Sox9 (ab185966, 1:100), CD63, (Santa Cruz, s-15,363, 1:200), LH3 (Proteintech, 11027-1-AP, 1:250), Vinculin (Sigma Aldrich,V9264, 1:500), gamma-H2AX

(Cell Signaling, 2577,1:200) diluted in the blocking solution overnight at 4°C. Cells were then washed and incubated in the dark with the fluorophore conjugated secondary antibody (Donkey anti-mouse/ rabbit, A10036/ A10040 ,1:400). Cells were incubated with Phalloidin (Invitrogen, A30107, 1:400), diluted in PBS, post-secondary antibody staining. The nuclear staining was obtained with DAPI (Sigma Aldrich, D9542) at room temperature. Coverslips were mounted onto slides using Fluoromount-G (Invitrogen, 00-4958-02) and visualized using Z-stacks of ECM and VSMCs was acquired using the Nikon Eclipse Ti Inverted Spinning Disk Confocal System. Validation of genuine target staining was determined using secondary antibody only negative controls. The images and fluorescence intensity were analysed by calculating the corrected total cell fluorescence (CTCF) on FIJI. ECM topology images were generated by the FIJI plugin Surface Plot. Colocalization percentage for nuclear DAPI and Sox9 was conducted by the FIJI plugin JaCoP (Just Another Colocalisation Plugin).

**Analysis of fibronectin and VSMC alignment:**

ECM fiber and cellular alignment analysis was performed using the Alignment by Fourier Transform (AFT) tool as previously described[34]. This algorithm divides inputted images into a grid of overlapping windows of a specified size, employing Fast Fourier Transforms (FFT) on each window to construct a field of orientation vectors representing the entire image. An alignment score or 'order parameter' is calculated for each vector by correlating its orientation with the orientations of the other vectors in a surrounding neighbourhood of a specified radius, using the equation $S = 2\left(< cos^2\theta_{ij} > -\frac{1}{2}\right)$, where $\theta_{ij}$ is the angle between the orientation of the central vector and its neighbours. The order parameter for each vector can range from -1 to 1, where -1 represents completely orthogonal alignment, 0 represents random alignment, and 1 represents completely parallel alignment. The median order parameter for each image is used to represent its overall alignment. ECM and VSMCs were visualised by staining for fibronectin and vinculin respectively. To analyse fibronectin

images with sufficient sensitivity to detect the local structural variation in the ECM meshwork, while maintaining comparison of fibre alignment over the greatest possible area, a small window of 50 px and large neighbourhood radius of 7 were used. Conversely, in VSMC images where granularity is comparatively low, a large window of 250 px and small neighbourhood radius of 1 were utilised, in order to capture the directionality only of whole cells and not finer structures or edges. Window overlap was 50% in all analyses.

**Edu Cell Proliferation:**

To measure cell proliferation, VSMCs were depleted from FBS for 24h to restart the cell cycle. Following depletion, cells were incubated with 10uM Click-iT Edu (ThermoFisher, C10337) for 1 hour in 20% FBS, 1% PSG supplemented M199 media. Edu visualization was achieved according to manufacturer protocol prior to any further immunofluorescence staining.

**CD63 Beads Assay:**

Quantification of CD63 positive sEV secretion was performed by the CD63 bead assay and flow cytometry analysis as previously described [31]. $1x10^8$ 4µm aldehyde-sulphate beads (Invitrogen, A37304) were added to filter sterilised 150µl MES buffer (Sigma, 76039). Beads were spun for 10 minutes at 3000g and washed twice more with MES buffer. Beads were incubated with 35µg of CD63 antibodies (BD Biosciences, 556019) overnight at room temperature with shaking. The beads were washed thrice with 4% filter sterilised BSA-PBS and resuspended in storage buffer (0.1% Glycine, 0.1% $NaN_3$ in PBS). Young and senescent VSMC were plated at a concentration of 15,000 cells per well. The following day they were washed thoroughly to remove residual exosomes and transduced with the control EGFP, Sox9 overexpression and, knockout adenovirus in 2.5% exosome-free FBS M199 media for three days. The media was then collected, spun for 5 minutes at 2500g and transferred to a new 24-well plate. The bead stock solution was washed once with 2% BSA-PBS, with 1µL of

bead suspension per each media sample. Beads were incubated in the media overnight at 4°C with gentle rocking. Cells were trypsinized and incubated with Solution13 (ChemoMetec, 910-3013) for a cell viability assay using the NC3000 cell nucleocounter (ChemoMetec). The beads were washed twice with 2% BSA-PBS and incubated with phycoerythrin (PE)-conjugated light sensitive anti-CD81 (BD Pharmigen, 555676, 1:50) antibody for 1 hour at room temperature. The beads were quantified using FACs analysis with a BD Accuri C6 flow cytometer. Data analysis was conducted using FlowJo software, with the number of sEVs normalised to the cell number that was calculated during the cell viability assay.

**Proteomics and bioinformatic analysis:**

Decellularized ECM protein samples were de-glycosylated as previously described [35]. Samples were precipitated overnight in acetone at -20°C and the protein pellet airdried in a speed vacuum. They were then incubated with chondroitinase (1:100) (Sigma, C3667), heparinase (1:500)( Sigma, H6512), keratanase (1:500) (Sigma, G6920), and 3 de-branching enzymes +1 O-deglycosidase (1:200 each) (EMD Millipore, 362280) diluted in the de-glycosylation buffer (Tris 200mM, NaAc 200mM, EDTA 100mM) at 25°C for two hours and then at 37°C overnight with shaking. Samples were air dried once again and incubated with PNGaseF (1:100) (Sigma Aldrich, 362185) diluted in $H_20^{18}$ for 48 hours at 37°C with shaking. Sample purification and trypsin digestion was then undertaken for peptide extraction. Samples were injected to be analyzed by LC-MS/MS in triplicate. Raw mass spectrometry data was processed into peak list files using Proteome Discoverer (ThermoScientific, v2.5) and processed using Sequest (Eng et al; PMID 24226387) search algorithms against the Uniprot Human Taxonomy database with a 1% FDR stringency. The overall intensity value for each peptide was compared across the sample set to further calculate changes in protein abundance. The mass spectrometry proteomics data have been deposited to the ProteomeXchange Consortium via the PRIDE[36] partner repository with the dataset identifiers PXD046001 and 10.6019/PXD046001.

Differentially expressed proteins were identified using DEP and Limma packages in R (Fold change set to 1, minimum P-value 0.05). The David tool was used to perform functional enrichment analysis on the lists of upregulated and downregulated proteins using cellular component terms from the Gene Ontology database. Corresponding dot plot graphs using the ggplot2 R package displaying fold enrichment, number of matched proteins in each cellular compartment term and Benjamini-Hochberg corrected q-value (linear blue-red colorscale) is presented. Proteins belonging to the classification of "extracellular space", "extracellular region", "basement membrane", and "extracellular matrix" were extracted, pooled together, and further sub-classified in Cytoscape app 3.8.0 with GeneMANIA app. The same process was repeated for all proteins initially classified under "extracellular exosome". Protein-protein interaction maps were built in Cytoscape, with proteins exhibiting minimal interactions with the network being discarded.

**Atomic Force Microscopy:**

Atomic Force Microscopy (AFM) data acquisition was done on a JPK Nanowizard 3. For AFM imaging, a tetrahedral shaped cantilever with a silicone tip with a spring constant of 0.081 M/m, tip height of 4-5μm (HYDRA-6v-200WG, Applied NanoStructures, CA) was used. The matrix stiffness was recorded via Contact Mode, measuring the force spectroscopy, in liquid. The set point was 5nN with a Z-length of 14 microns and extend speed of 10μm/second. As the synthesized ECM thickness was uniform across the coverslip, areas for measurement were chosen at random. The AFM data was processed via the JPK Nanowizard 3 data processing software.

**Vesicle Isolation:**

EVs were isolated and purified by ultracentrifugation (UC) as previously described [31]. VSMCs were washed twice with Earl's balanced salt solution (EBSS) and the media changed to Dulbecco's modified eagle medium (DMEM) (Sigma, D5921) supplemented with 0.1% BSA and 1% PSG. After 24h the media was collected and centrifuged at 1000g to

pellet apoptotic bodies, at 10,000g to pellet micro-vesicles, and 100,000g by UC to pellet small EVs. Pellets were washed and resuspended in PBS.

**Statistical Analysis:**

All results are from three biological replicates utilising different VSMC donor isolates. All results are presented as +/-Standard error of mean (SEM). Statistical analysis was performed in GraphPad software. Normality tests were conducted for all datasets via the Shapiro-Wilk test prior to comparison analysis. For data with less than 10 samples where the normality test could not be conclusive, we have presented evidence from prior larger sample size studies that have proven normality for RNA expression or protein abundance of Sox9[23,37], p16[38,39], p21[38,39], IL6[40,41] and Plod3/LH3[42,43]. If normality was validated, Multiple groups were compared via 2-way ANOVA, adjusting for multiple testing with Benjamini Hochberg FDR correction, whilst two independent groups were compared via the Student *t* test. If normality was rejected, multiple groups were compared via Mixed Effect Analysis, with multiple testing correction via FDR method of Benjamini and Hochberg. Two independent non-normally distributed groups were compared via the Mann-Whitney test. Pearson's (parametric) test was applied to determine the p value and R squared fit between two different proteins in histological samples.

| Age | Gender | AHA classification of atherosclerotic plaque | Calcification |
|---|---|---|---|
| 18 | M | I | - |
| 19 | M | I | - |
| 23 | M | I | + + |
| 30 | F | III | - |
| 34 | M | II | + + + + |
| 38 | F | II | + |
| 39 | M | III | +++ |
| 41 | M | II | + + + |
| 44 | M | II | - |
| 45 | M | III | - |
| 47 | M | III | + + + + |
| 49 | F | III | +++ |
| 50 | M | IV | + |
| 55 | F | II | + + + |
| 58 | M | III | + + + + |
| 63 | F | IV | + + + + |
| 66 | M | II | - |
| 70 | M | IV | ++ |
| 84 | F | III | + + + + |
| 85 | F | IV | - |
| 89 | F | IV | + + |

S Table 1: Table describing the age, gender, American Heart Association (AHA) classification of atherosclerotic plaques, and level of calcification within the Medial aortic area. Lack of calcification is denoted by - , whist severity of calcification is graded from + to + + + +.

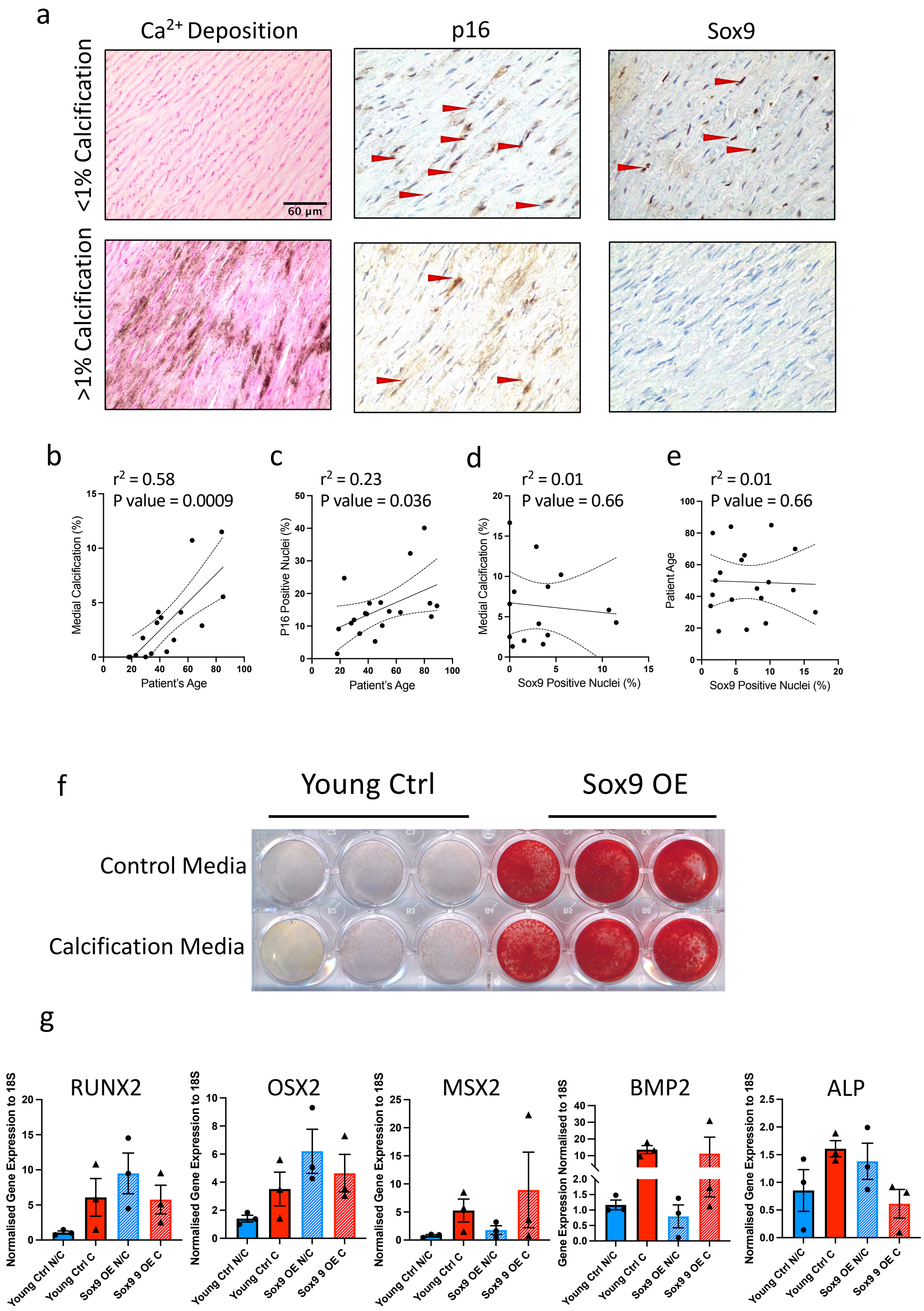


S Fig1: Sox 9 does not associate with vascular calcification. (a) Representative images of matched patient tissue samples stained for Von Kossa (Calcification), Sox9, and p16. Red arrows indicate prominent positive cellular staining. Correlation between calcium deposition (Medial Calcification %), age, cellular senescence (p16), and Sox9 expression. Correlation between (b) Medial Calcification and Patient Age (n=15), (c) p16 and Patient's age (n=19) (d) Medial Calcification and positive Sox9 nuclei (%) (n=16), and (e) Sox9 and patient age (n=19). Normality was validated via Shapiro-Wilk. Statistical significance determined via Pearson's Correlation. (f) Alizarin red staining showing Sox9 did not increase calcification of VSMCs *in vitro* (n=3 isolates). (g) Gene expression of calcification markers, quantified via RT-qPCR, showing Sox9 expression did not change osteogenic gene expression in calcifying VSMCs (n=3 isolates). Normality validated via Shapiro-Wilk, One-way ANOVA showed no significance.

S. Figure 1

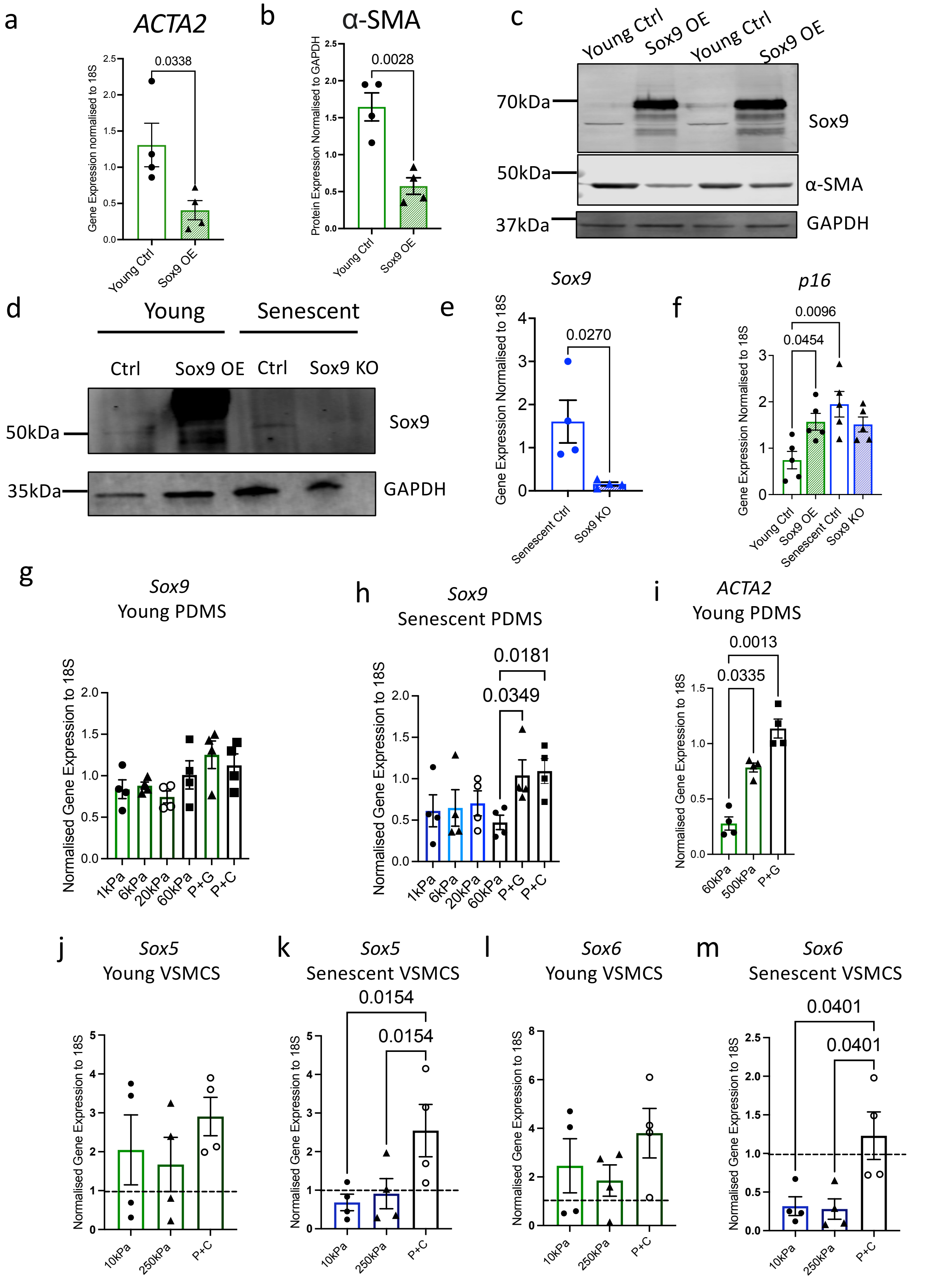


S Fig2: Sox9 regulates Vascular Smooth Muscle Cell Phenotype. (a) Gene expression, quantified via RT-qPCR of ACTA2, in young VSMCs transfected with control EGFP adenovirus (Young Ctrl) and Sox9 overexpressing adenovirus (Sox9 OE), (n=4 from 3 isolates). Normality validated via Shapiro-Wilk, Unpaired Student's T-test. (b,c) Quantification of protein expression and Western blot of α-SMA from young Ctrl and Sox9 OE cell lysate samples (n=4 from 3 isolates). Normality validated via Shapiro-Wilk, Unpaired Student's T-test. (d) Western blot of Sox9 expression in young Ctrl, Sox9 OE, senescent VSMCs transduced with shEGFP control adenovirus (Senescent Ctrl), and Sox9 knockout adenovirus (Sox9 KO). (e) Gene expression of Sox9 in senescent VSMCs transduced with control shEGFP adenovirus (Senescent Ctrl) and Sox9 knockout (Sox9 KO) adenovirus (n=4 from 3 isolates). Normality validated via Shapiro-Wilk, Unpaired Student's T-test. (f) Gene expression of p16 in Young Ctrl, Sox9 OE, Senescent Ctrl, and Sox9 KO (n=4 from 3 isolates). Sox9 gene expression in (g) young and (h) senescent VSMCs when plated on PDMS gels of various stiffnesses. (i) ACTA2 gene expression in young VSMCs plated on PDMS gels. Normality validated via Shapiro Wilk, Two-Way ANOVA with FDR Corrections, q-values presented. Gene expression of Sox5 in (j) young and (k) senescent VSMCs plated on matrices of different stiffness and plastic coated with collagen (P+C). Gene expression of Sox8 of (l) young and (m) senescent VSMCs plated on hydrogels of 10kpa and 250kpa, and P+C (n=4 from 3 isolates). Genes normalized to young and senescent VSMCs plated on plastic (indicated by the dotted line). Normality validated via Shapiro-Wilk, 2way ANOVA, q-values adjusted for multiple testing with Benjamini Hochberg FDR correction.

S. Figure 2

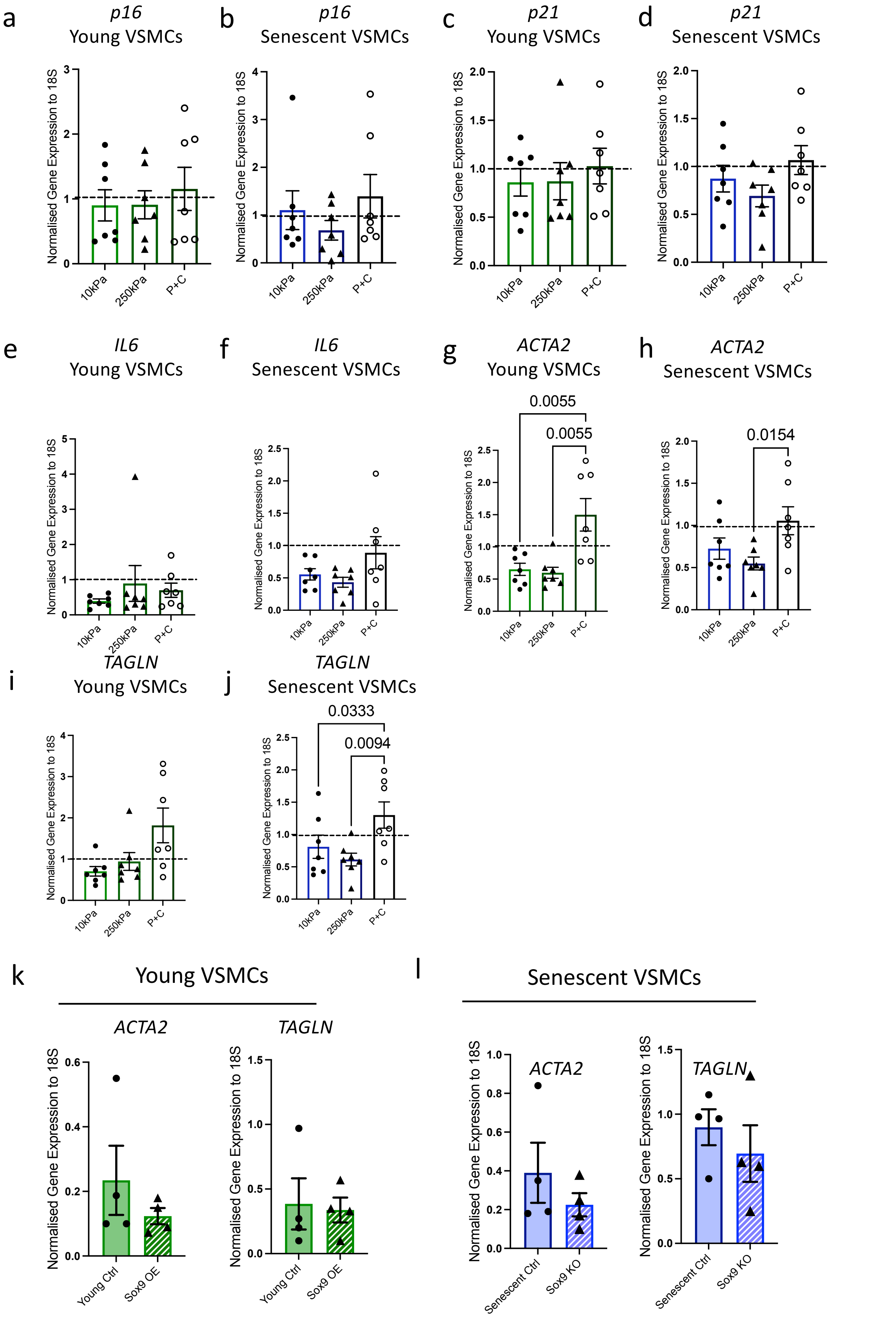


S. Figure 3: (a-b) p16, (c-d) p21, (e-f) IL6, (g-h) ACTA2, and (i-j) TAGLN gene expression, quantified via RT-qPCR, in young and senescent VSMCs on 10kPa and 250kPa hydrogels and plastic coated with collagen (P+C) (n=7 from 3 isolates). Normality validated via Shapiro-Wilk, 2-way ANOVA with q-values adjusted for multiple testing with Benjamini Hochberg FDR correction . (k-l) ACTA2 and TAGLN gene expression in VSMCs on ECM synthesized from young VSMCs treated with control (Young Ctrl) or Sox9-overexpressing (Sox9 OE) adenovirus, and senescent VSMCs treated with control (Senescent Ctrl) or Sox9 knockout (Sox9 KO) adenovirus (n=4 from 3 isolates). Normality rejected (ACTA2 young VSMCs) or accepted via Shapiro-Wilk. Mann-Whitney (Normality rejected ) or Unpaired Student's T-test (Normality accepted) statistical tests showed no significance.

S. Figure 3

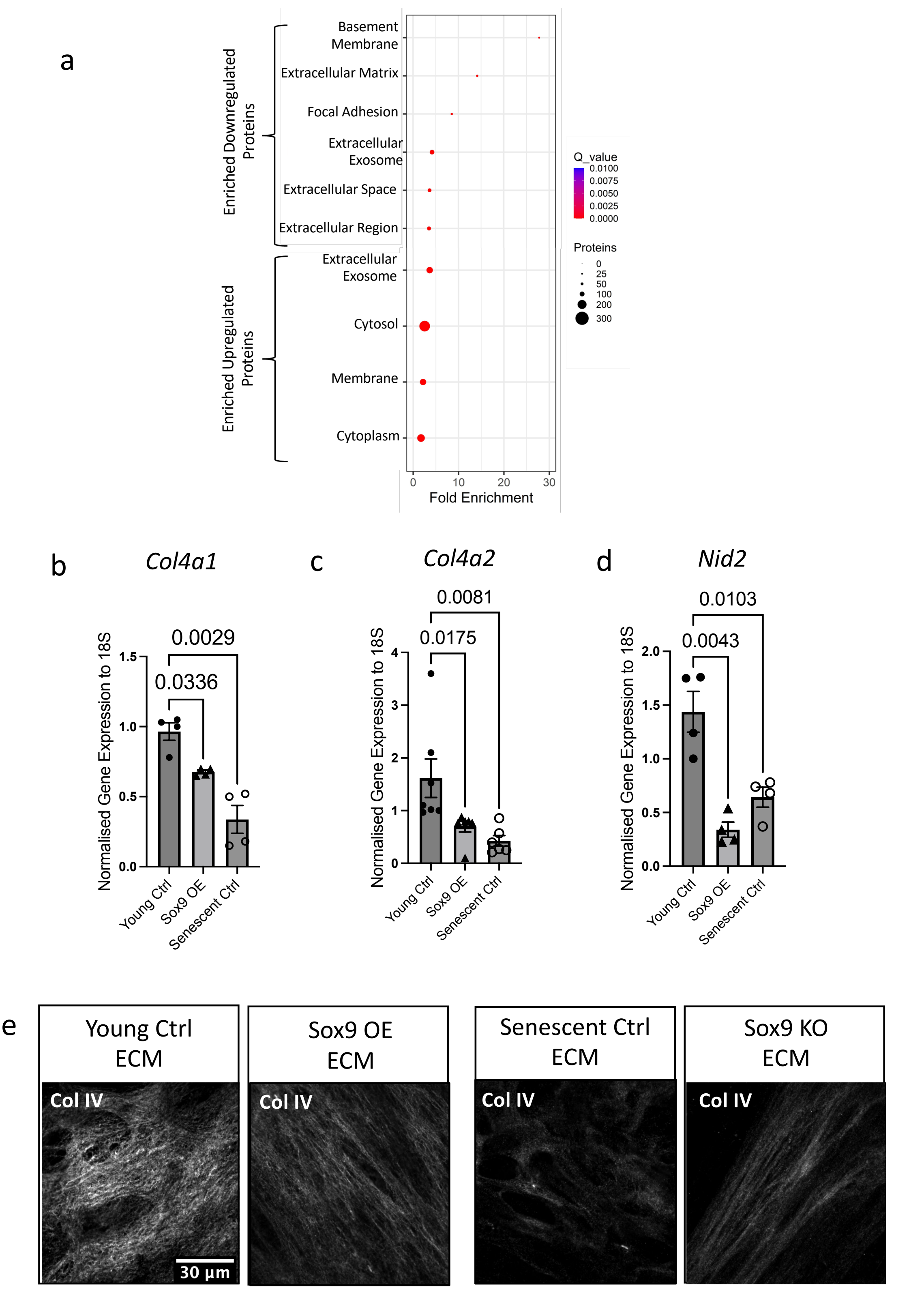


S Figure 4: (a) GO analysis showing pathways regulated by differentially expressed genes between young and senescent VSMCs. Gene expression, quantified via RT-qPCR of (b) Collagen (Col)4a1, (c), Col4a2, and (d) nidogen2 (Nid2) from young VSMCs transduced with EGFP control adenovirus (Young Ctrl), Sox9 overexpression adenovirus (Sox9 OE), and senescent cells transduced with shEGFP control adenovirus (Senescent Ctrl) (n=4 from 3 isolates). Normality either rejected (Col4a1) or validated (col4a1, Nid2) via Shapiro-Wilk, Mixed effect model (normality rejected) or 2-way ANOVA (normality accepted), with q-values adjusted for multiple testing with Benjamini Hochberg FDR correction.(e) Immunofluorescence of Collagen 4 (Col IV) in ECM synthesized from Young Ctrl, Sox9 OE, Senescent Ctrl, and senescent VSMCs transduced with Sox9 knockout adenovirus (Sox9 KO).

S. Figure 4

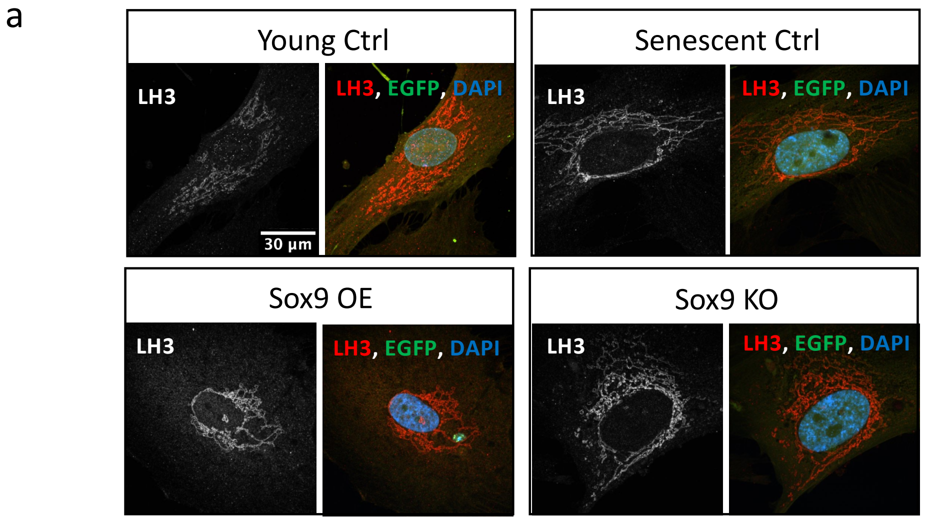


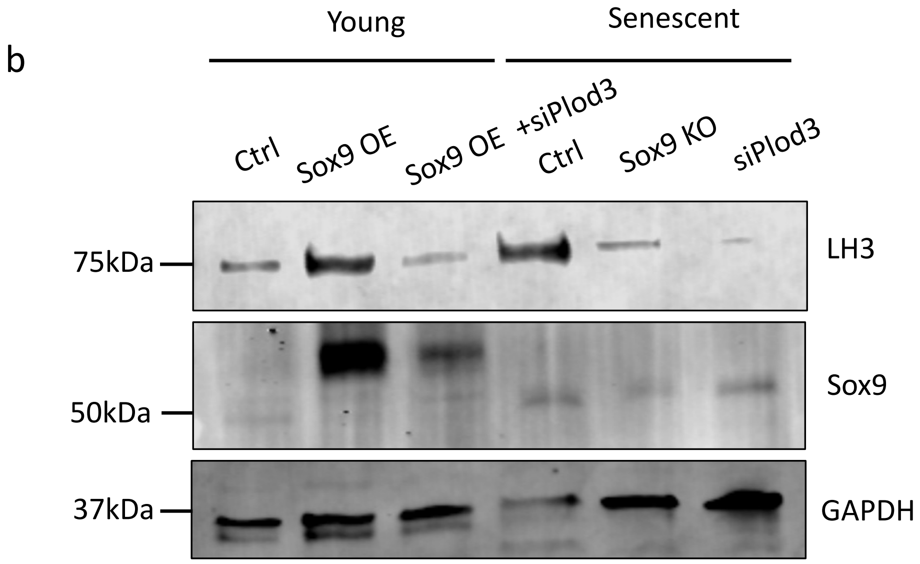


S Figure 5: (a) Representative immunofluorescence images of LH3 in young VSMCs transduced with EGFP control adenovirus (Young Ctrl), Sox9 overexpression (Sox9 OE), and senescent VSMCs transduced with shEGFP control adenovirus (Senescent Ctrl), and Sox9 knockout (Sox9 KO). LH3 is in grey and red, EGFP in green, and nuclear staining (DAPI) in blue. (b) Western blot depicting LH3 knockout (siPlod3) with Sox9 overexpression (Sox9 OE) and Sox9 knockout (KO) from protein lysate in young and senescent Vascular Smooth Muscle Cells.

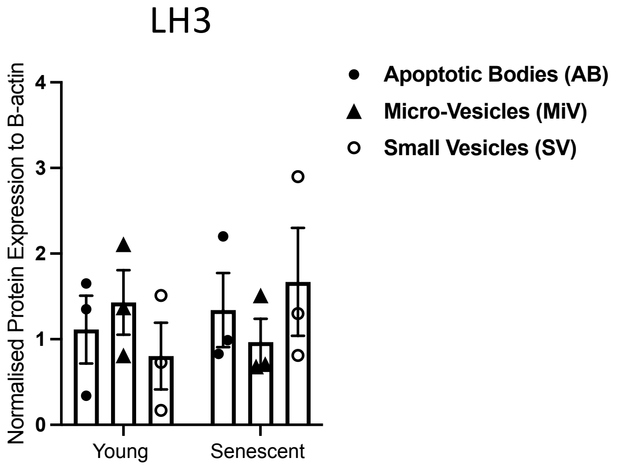


S. Figure 6: Protein quantification of LH3 from three types of extracellular vesicles in young and senescent Vascular Smooth muscle cells (n=3 isolates). Normality accepted via Shapiro-Wilk, 2way ANOVA showed no significance.
